# Supplementary figures and images for: Molecular circadian rhythms are robust in marine annelids lacking rhythmic behavior
Source: PLoS Biol. 2024 Apr 11;22(4):e3002572. doi: 10.1371/journal.pbio.3002572 (PMC11008795; doi:10.1371/journal.pbio.3002572)

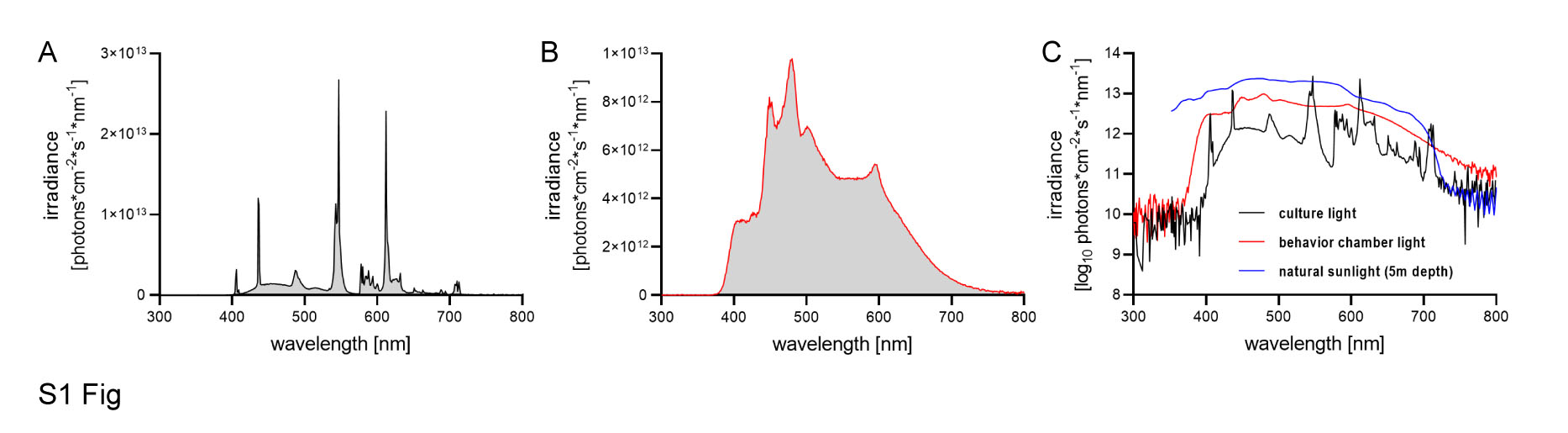

Supplement: S1 Fig — (A) Standard worm culture light spectrum. (B) Behavior chamber light spectrum. (C) Logarithmic plotting of panels A and B as well as a natural sunlight spectrum recorded in the natural habitat of Platynereis dumerilii around Ischia, Italy, at 5 m depth in November 2011 (10 AM–4 PM local time average) [38]. Overall irradiance (380–750 nm) was 4.78 * 1014 photons * cm−2 * s−1 for the worm culture and 1.40 * 1015 photons * cm−2 * s−1 for the behavior chamber. (JPG) [file pbio.3002572.s001.jpg]

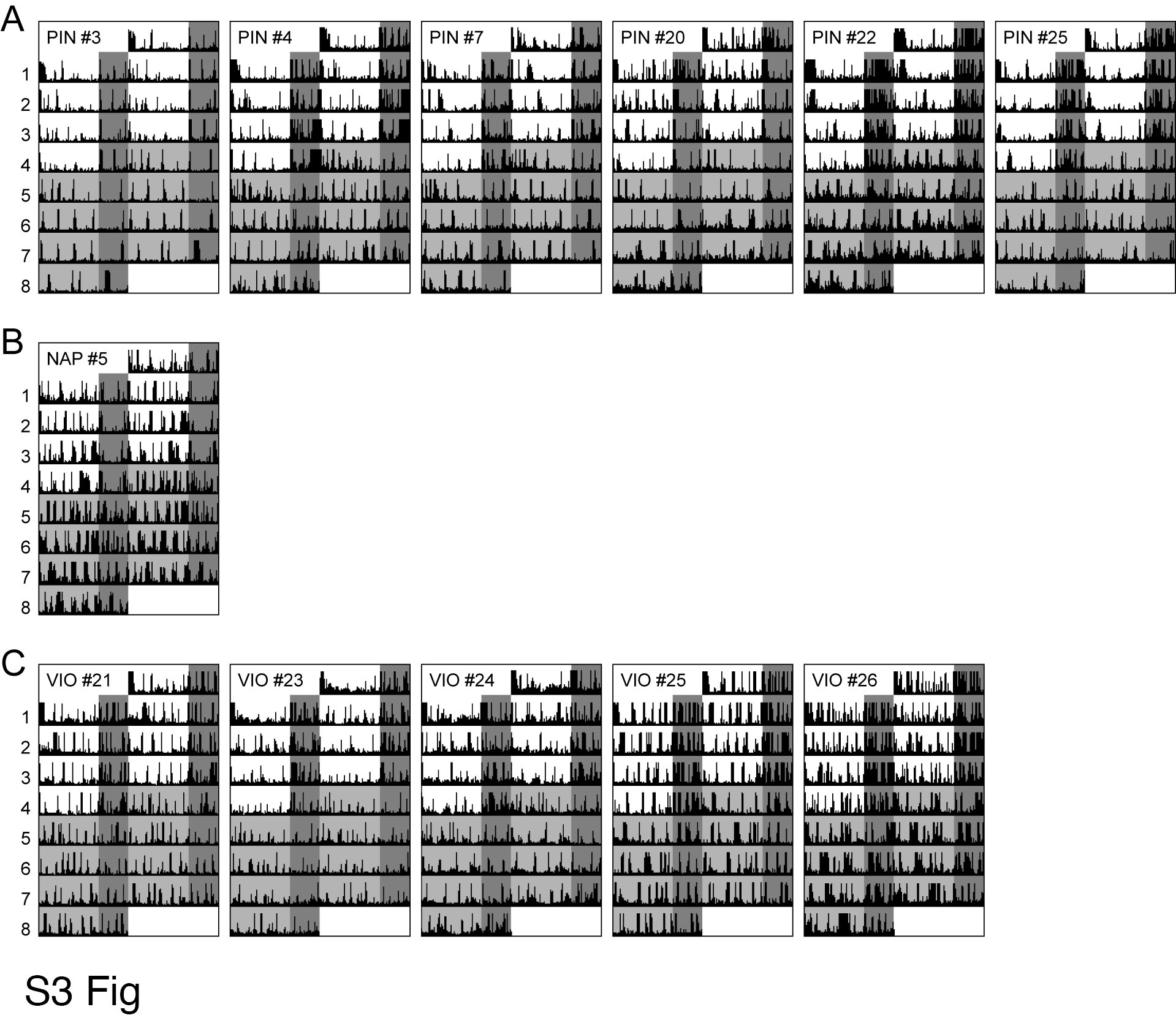

Supplement: S3 Fig — Related to Figs 1, S2 and S4. Double-plotted actograms of individual (A) PIN, (B) NAP, and (C) VIO wild-type worms are shown. Locomotor activity was recorded over 4 d of LD (16 h:8 h) and 4 d of DD. Y-axis is magnified 10-fold relative to S2 Fig to better visualization activity patterns. The comparison illustrates that although arrhythmic worms showed overall lower activity, they were far from inactive. Individual worm identifiers (#) match those in Figs S2 and S4. (JPG) [file pbio.3002572.s003.jpg]

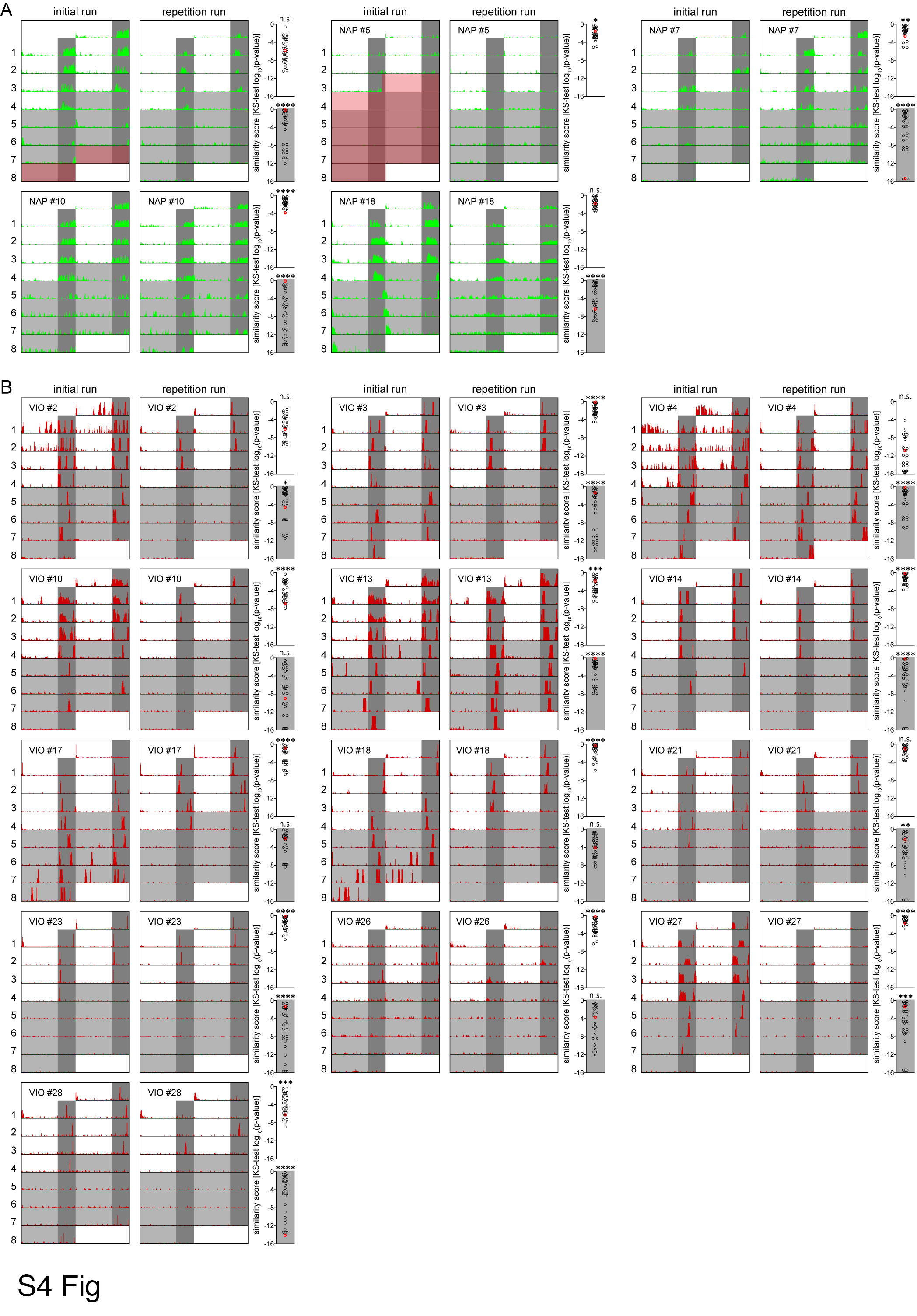

Supplement: S4 Fig — Related to Fig 1. Double-plotted actograms of (A) NAP strain and (B) VIO strain individual worms are shown. Locomotor behavior was recorded over 4 d of LD (16 h:8 h) and 8 d of DD in 2 consecutive runs (initial/repetition). #: individual worm identifier. Red shading indicates that worms crawled out of the tracking well. Worms that were excluded from statistics due to maturation during or within 1 week after the recording are not shown, as maturation strongly alters their overall behavior. For an explanation of scatter plots on behavioral similarity in LD and DD (gray background), see Fig 1E. In the VIO strain (B), 7/13 individual worms in both LD and DD showed significantly higher similarity for matching initial/repetition runs. There were also a few cases where an initial run was significantly less similar to the matching repetition run than to the control group (LD: 3/13, DD: 2/13). NAP strain individuals (A) showed little behavioral reproducibility with some worms showing significantly higher similarity for matching initial/repetition runs (LD: 1/5, DD: 2/4), while other worms it was significantly lower (LD: 2/5, DD: 2/4). (JPG) [file pbio.3002572.s004.jpg]

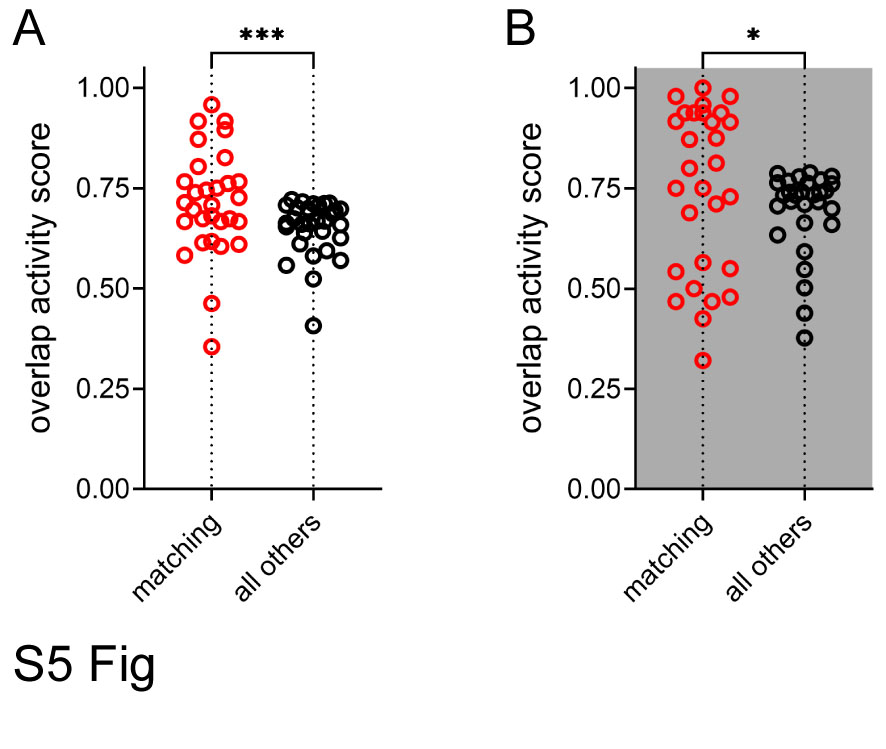

Supplement: S5 Fig — Related to Figs 1E and S4. Similarity (overlap activity scores) of initial runs against matching repetition runs, and against mean values of all other (nonmatching) repetition runs were compared via Wilcoxon matched-pairs signed-rank test for LD (A) and DD (B, gray background). Significance levels: *p < 0.05, **p < 0.01, ***p < 0.001. (JPG) [file pbio.3002572.s005.jpg]

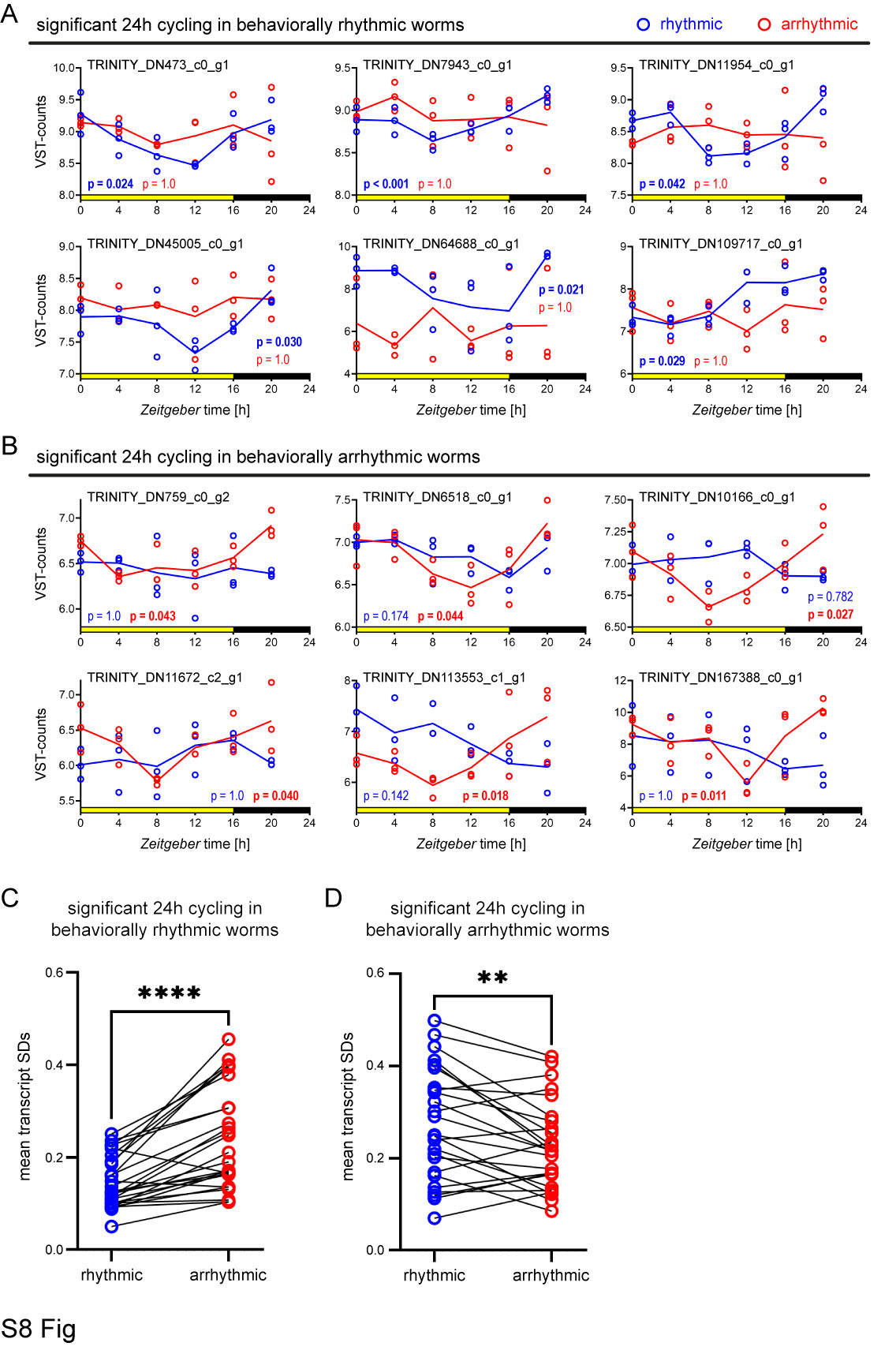

Supplement: S8 Fig — arrhythmic worms. Related to Fig 3. Representative transcripts with significant 24-h cycling only rhythmic worms (A) or only in arrhythmic worms (B) were selected at random from the bottom of the heatmaps in Fig 3B and 3D and are plotted here (rhythmic: blue, arrhythmic: red). Bold p-values (FDR-corrected) indicate significant 24-h cycling. Per time point, n = 3 replicates were measured. (C) Transcripts with significant 24-h cycling only in behaviorally rhythmic worms. The used transcripts (n = 26) are associated with the GO-terms “neuromuscular process controlling balance,” “axon regeneration,” “visual behavior,” and “response to hypoxia.” (D) Transcripts with 24-h cycling only in behaviorally arrhythmic worms. The used transcripts (n = 25) are associated with the GO-terms “fatty acid beta-oxidation using acyl-CoA dehydrogenase,” “glucose metabolic process,” “excretion,” “response to vitamin A,” “mitochondrial transmembrane transport,” and “phosphatidylinositol phosphorylation.” Mean transcript SDs for a given transcript and phenotype were calculated as mean of the SDs for the 6 individual time points (n = 3 samples per time point). Variance was compared between rhythmic (blue) and arrhythmic (red) phenotypes via paired 2-sided t test. Black lines indicate value pairs belonging to the same transcript. Significance levels: *p < 0.05, **p < 0.01, ***p < 0.001, ****p < 0.0001. While there was a general trend of lower variance in the phenotype with cycling, in both (C) and (D), there are transcripts with no or the opposite trend. The higher variances for neuronal/behavioral transcripts (C) in behaviorally arrhythmic worms are fully consistent with the observed behavior. Detailed RAIN results are provided in S2E–S2G Table. (JPG) [file pbio.3002572.s008.jpg]

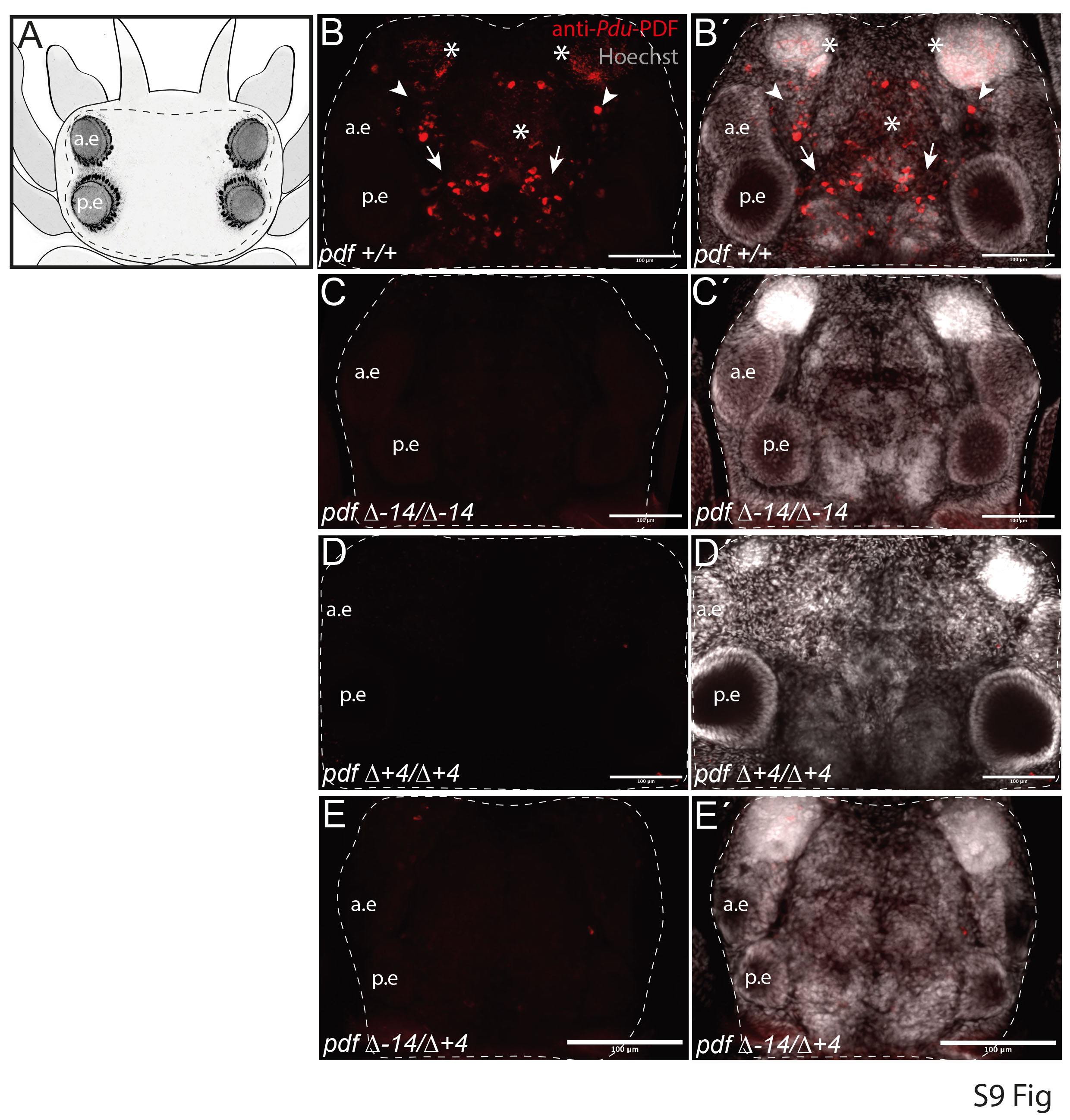

Supplement: S9 Fig — Related to Fig 5. Heads of immature worms were stained with anti-Pdu-PDF antibody, in combination with Hoechst staining of nuclei. (A) Schematic of P. dumerilii head. (B, B’) pdf +/+ (wild-type) heads show staining between the posterior eyes partially overlapping with the posterior oval-shaped domain (arrows) between the posterior eyes (p.e.) [37], adjacent to the anterior eyes (a.e. arrowheads), as well as in neural projections in the center of the head and mushroom bodies (*). (C-E’) Immunohistochemistry with anti-Pdu-PDF in worms carrying different combinations of mutant pdf alleles (Δ−14/Δ−14; Δ+4/Δ+4; Δ−14/Δ+4) showed that neither of the mutant worms have PDF staining. Scale bar: 100 μm. (JPG) [file pbio.3002572.s009.jpg]

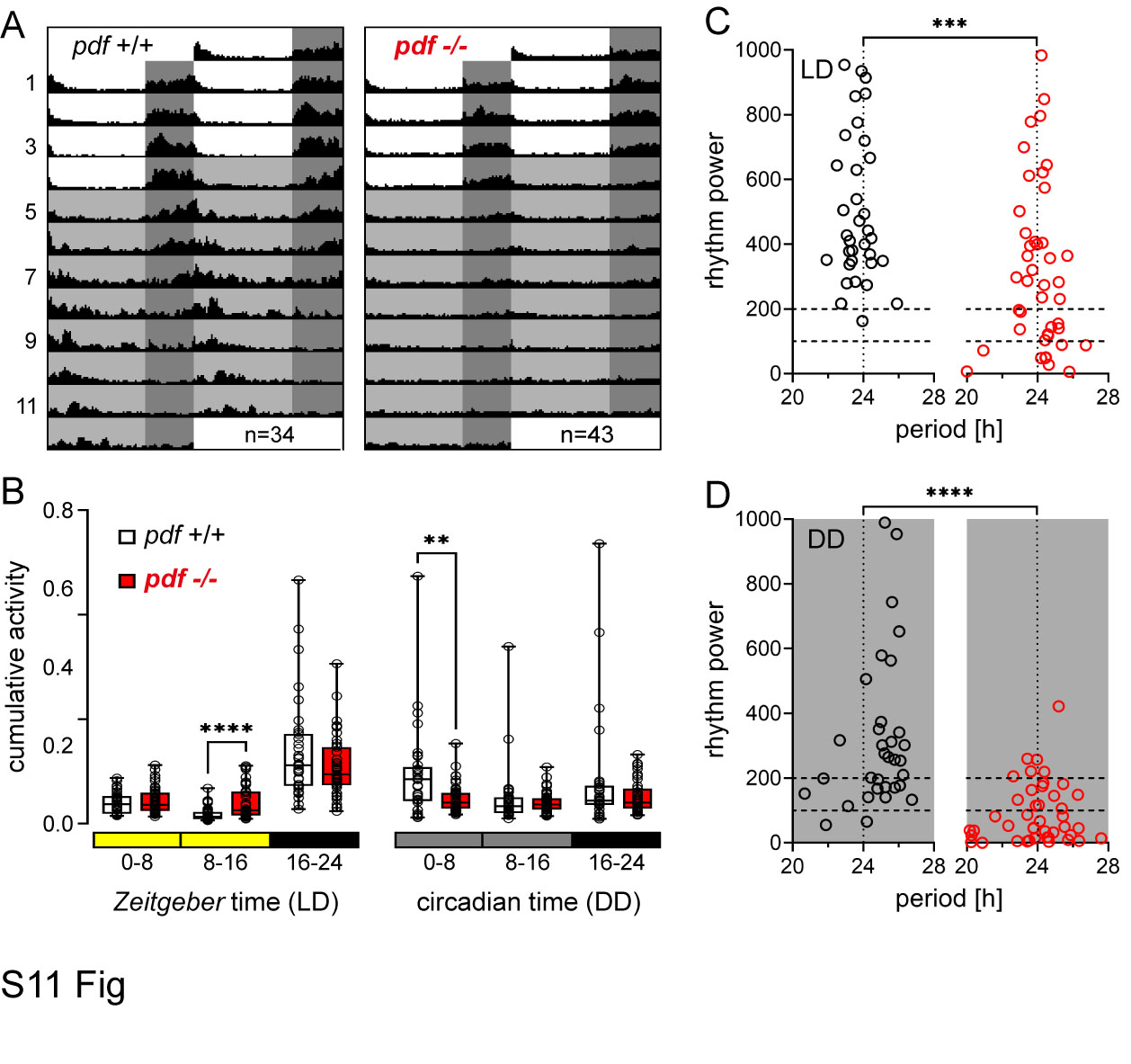

Supplement: S11 Fig — Related to Fig 6. In 4 recordings, n = 34 pdf wild types from 4 mating batches were compared to n = 43 pdf mutants from 5 mating batches (−14/−14 n = 33, −14/+4 n = 10). (A) Circadian locomotor activity of VIO strain pdf wild types (black) and mutants (red) under 4 d of LD and 8 d of DD. Individual worm actograms are provided in S10C and S10D Fig. (B) Cumulative activity over the early day (0–8), late day (8–16), and night (16–24) in LD and DD. (C, D) Period/power of wild type and mutant locomotor rhythms in the circadian range (20 h–28 h) in LD and DD determined by Lomb–Scargle periodogram. Statistical differences (panels B–D) were determined via Mann–Whitney U-test. Significance levels: *p < 0.05, **p < 0.01, ***p < 0.001, ****p < 0.0001. For period/power values, see S1D Table. For further info on figure labeling, see Fig 1. The reduced rhythmicity of pdf mutants disappeared directly after an outcross against the PIN strain (Fig 6) and could not be recovered by backcross against the VIO strain (S12 Fig). Hence, we consider it not causally connected to the pdf mutant locus. We attribute the difference of the VIO wild types here compared to the VIO strain worms in Fig 1 to the high polymorphism rate, which in marine invertebrates can reach 4%–5% in the maternal vs. paternal genomes of ONE individual [110]. (JPG) [file pbio.3002572.s011.jpg]

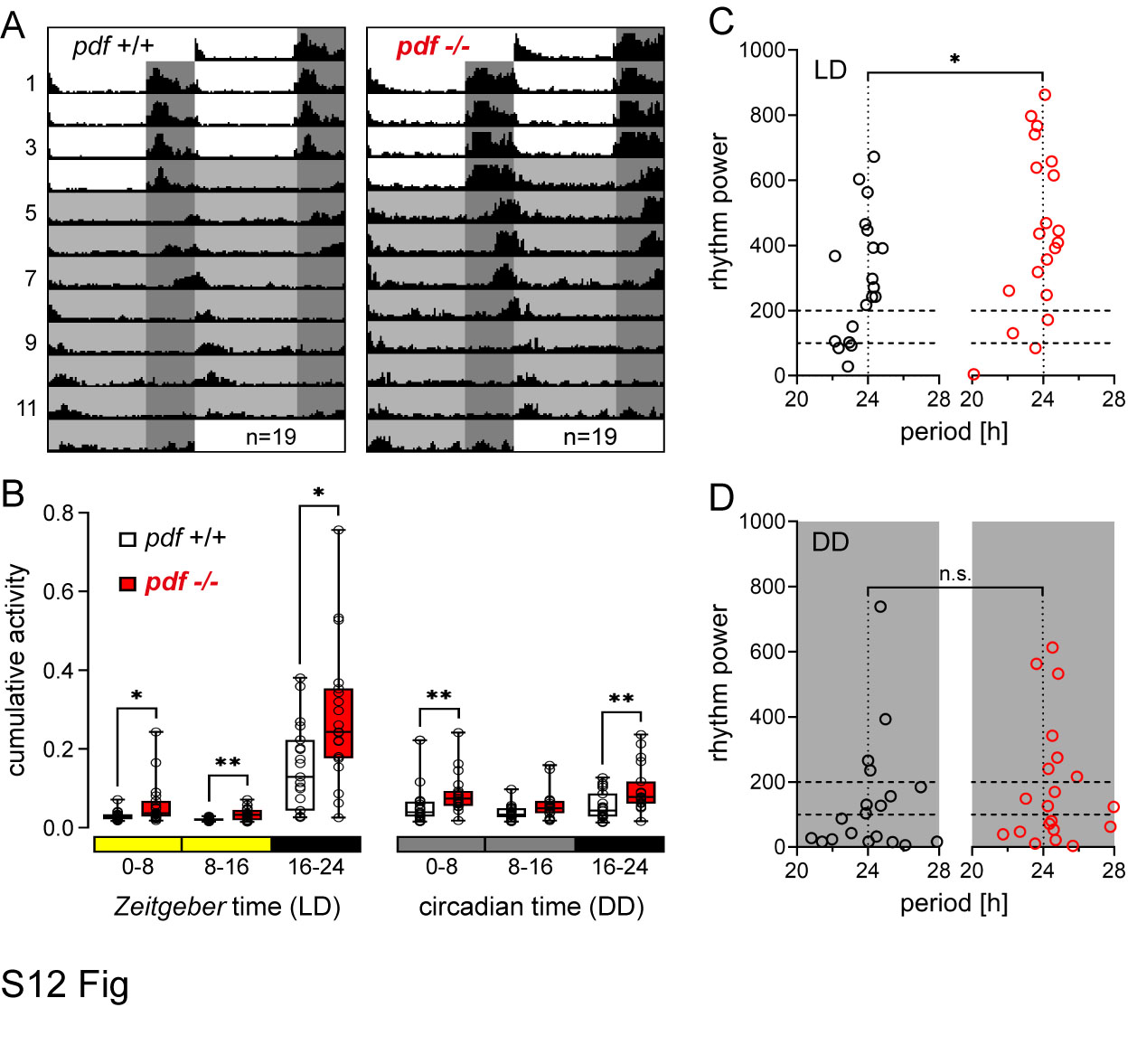

Supplement: S12 Fig — Related to Fig 6. In 1 recording, n = 19 pdf wild types from 5 mating batches were compared to n = 19 pdf mutants from 6 mating batches (−14/−14 n = 9, +4/+4 n = 10). (A) Circadian locomotor activity of VIO-backcrossed pdf wild types (black) and mutants (red) under 4 d of LD and 8 d of DD. Individual worm actograms were are provided in S10E and S10F Fig. (B) Cumulative activity over the early day (0–8), late day (8–16), and night (16–24) in LD and DD. (C, D) Period/power of wild type and mutant locomotor rhythms in the circadian range (20 h–28 h) in LD and DD determined by Lomb–Scargle periodogram. Statistical differences were determined via Mann–Whitney U-test (panels B and C) or unpaired 2-sided t test (panel D). Significance levels: *p < 0.05, **p < 0.01, ***p < 0.001, ****p < 0.0001. For period/power values, see S1E Table. For further info on figure labeling, see Fig 1. The persistence of the phenotype observed in VIO/PIN background worms (Fig 6) reinforces that this is a solid pdf mutant phenotype and that the initial rhythmicity reduction in the VIO strain (S11 Fig) is not causally connected to the pdf mutant locus. (JPG) [file pbio.3002572.s012.jpg]
