# Supplementary material for: Molecular circadian rhythms are robust in marine annelids lacking rhythmic behavior
Source: PLoS Biol. 2024 Apr 11;22(4):e3002572. doi: 10.1371/journal.pbio.3002572 (PMC11008795; doi:10.1371/journal.pbio.3002572)
Supplement: S2 Fig — Related to Fig 1. Double-plotted actograms of individual worms from the (A) PIN strain, (B) NAP strain, and (C) VIO strain are shown. Locomotor activity was recorded over 4 d of LD (16 h:8 h) and 8 d of DD. #: individual worm identifier. Red shading indicates that worms crawled out of the tracking well. Worms that maturated during or within 1 week after the recording were excluded from statistics and are not shown, as maturation strongly alters their overall behavior. For worms that matured later (used in statistics), sex is indicated, if known (not recorded systematically). Sexes were determined to check for potential sex-specific behavioral differences, of which none were found. (PDF) [file pbio.3002572.s002.pdf]

<u>S2 Fig: Individual worm actograms of strain comparison.</u> Related to Fig 1. Double-plotted actograms of individual worms from the (A) PIN strain, (B) NAP strain and (C) VIO strain are shown. Locomotor activity was recorded over 4 LD days (16h:8h) and 8 DD days. #: individual worm identifier. Red shading indicates when worms crawled out of the tracking well. Worms that maturated during or within one week after the recording were excluded from statistics and are not shown, as maturation strongly alters their overall behavior. For worms that matured later (used in statistics) sex is indicated, if known (not recorded systematically). Sexes were determined to check for potential sex-specific behavioral differences, of which no were found.

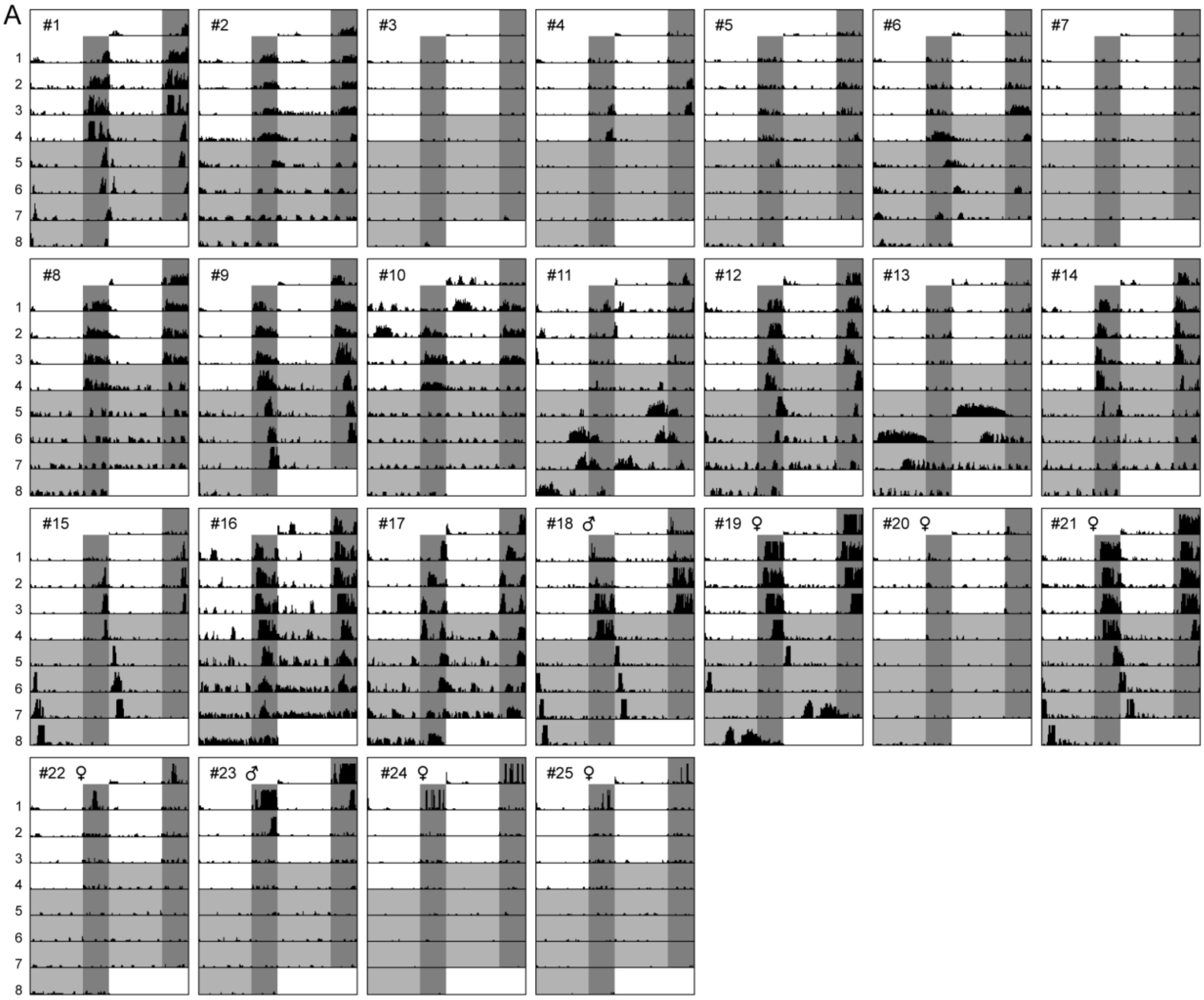

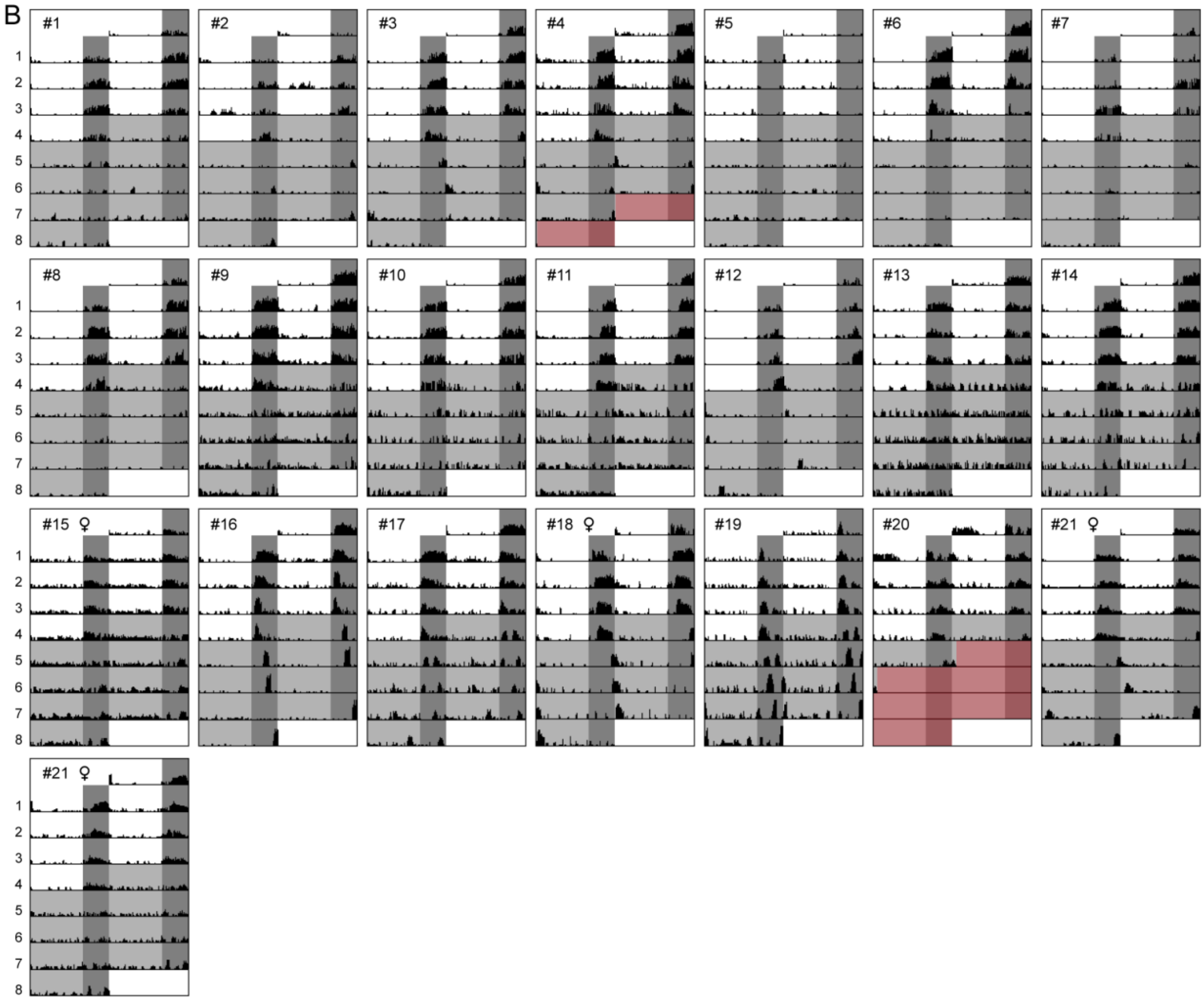
B
#1
#2
#3
#4
#5
#6
#7
#8
#9
#10
#11
#12
#13
#14
#15 ♀
#16
#17
#18 ♀
#19
#20
#21 ♀
#21 ♀
1
2
3
4
5
6
7
8

C

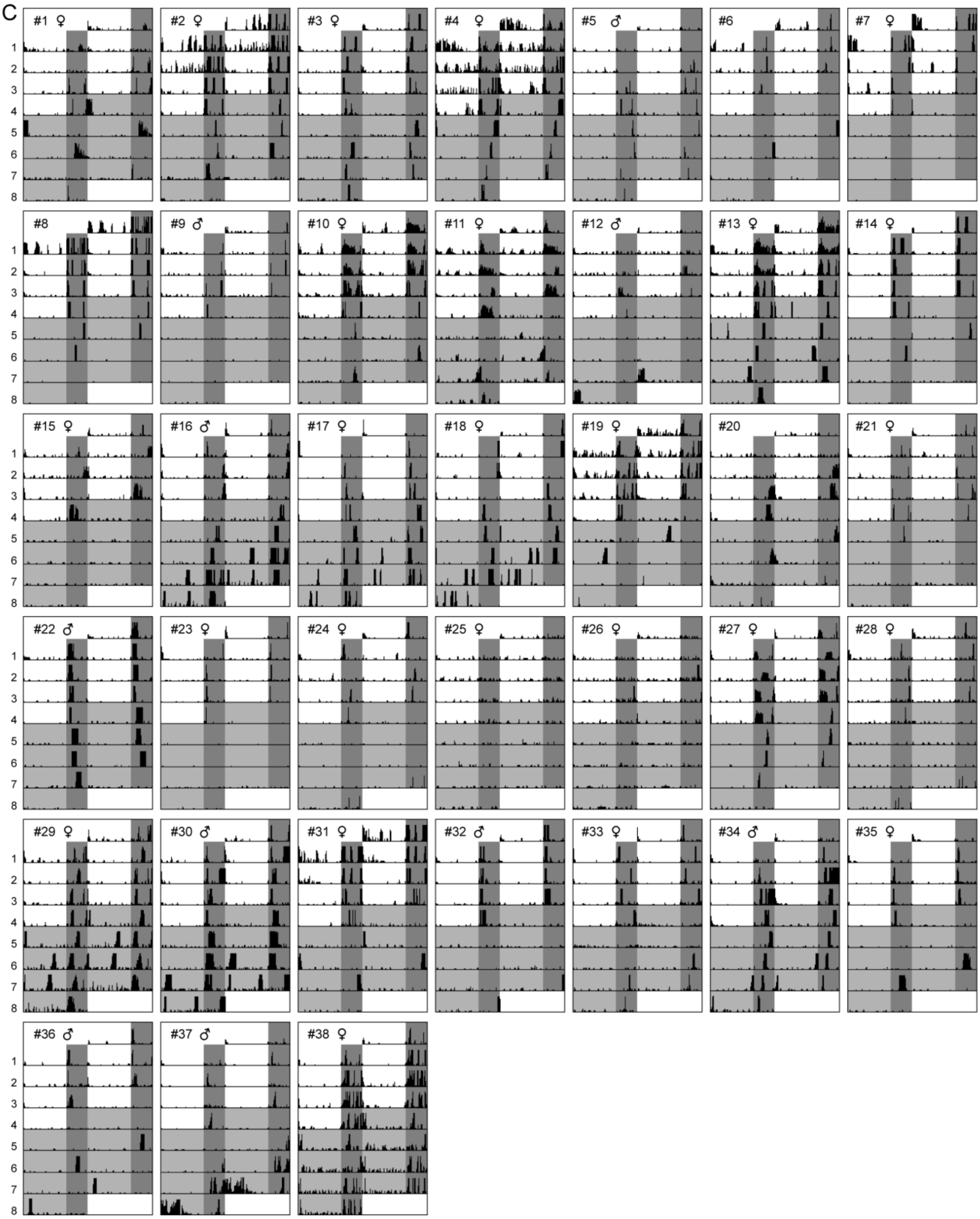
#1 ♀
#2 ♀
#3 ♀
#4 ♀
#5 ♂
#6
#7 ♀
#8
#9 ♂
#10 ♀
#11 ♀
#12 ♂
#13 ♀
#14 ♀
#15 ♀
#16 ♂
#17 ♀
#18 ♀
#19 ♀
#20
#21 ♀
#22 ♂
#23 ♀
#24 ♀
#25 ♀
#26 ♀
#27 ♀
#28 ♀
#29 ♀
#30 ♂
#31 ♀
#32 ♂
#33 ♀
#34 ♂
#35 ♀
#36 ♂
#37 ♂
#38 ♀
1
2
3
4
5
6
7
8
