# Supplementary material for: Molecular circadian rhythms are robust in marine annelids lacking rhythmic behavior
Source: PLoS Biol. 2024 Apr 11;22(4):e3002572. doi: 10.1371/journal.pbio.3002572 (PMC11008795; doi:10.1371/journal.pbio.3002572)

<u>S6 Fig: Individual actograms of worms characterized for RNASeq analysis.</u> Related to Fig 2. Double-plotted actograms of individual PIN wildtype worms are shown. Locomotor activity was recorded over 3 LD days (16h:8h) and 3 DD days. Per behavioral recording, 25 worms were investigated in parallel. Two identical behavior chambers were used for recordings. Each page contains all worms of a characterization run including worms that matured and were thus excluded. #: individual worm identifier. Letters indicate behavioral characterization as rhythmic (R), arrhythmic (A) or intermediate (i). Red shading indicates that worms crawled out of the tracking arena.

recording #1 (02.11.2019-07.11.2019, chamber 1)

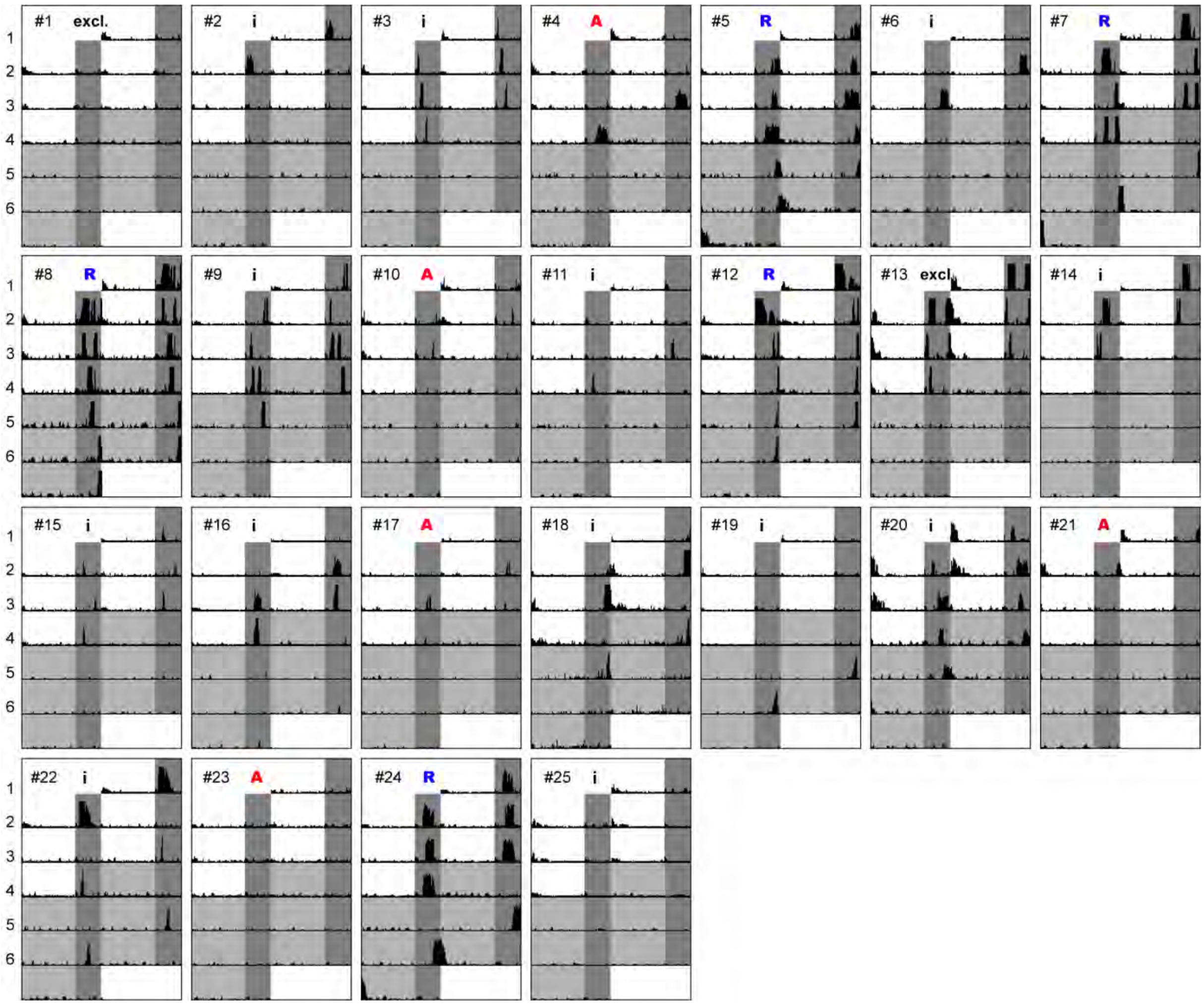

recording #2 (06.11.2019-13.11.2019, chamber 2)

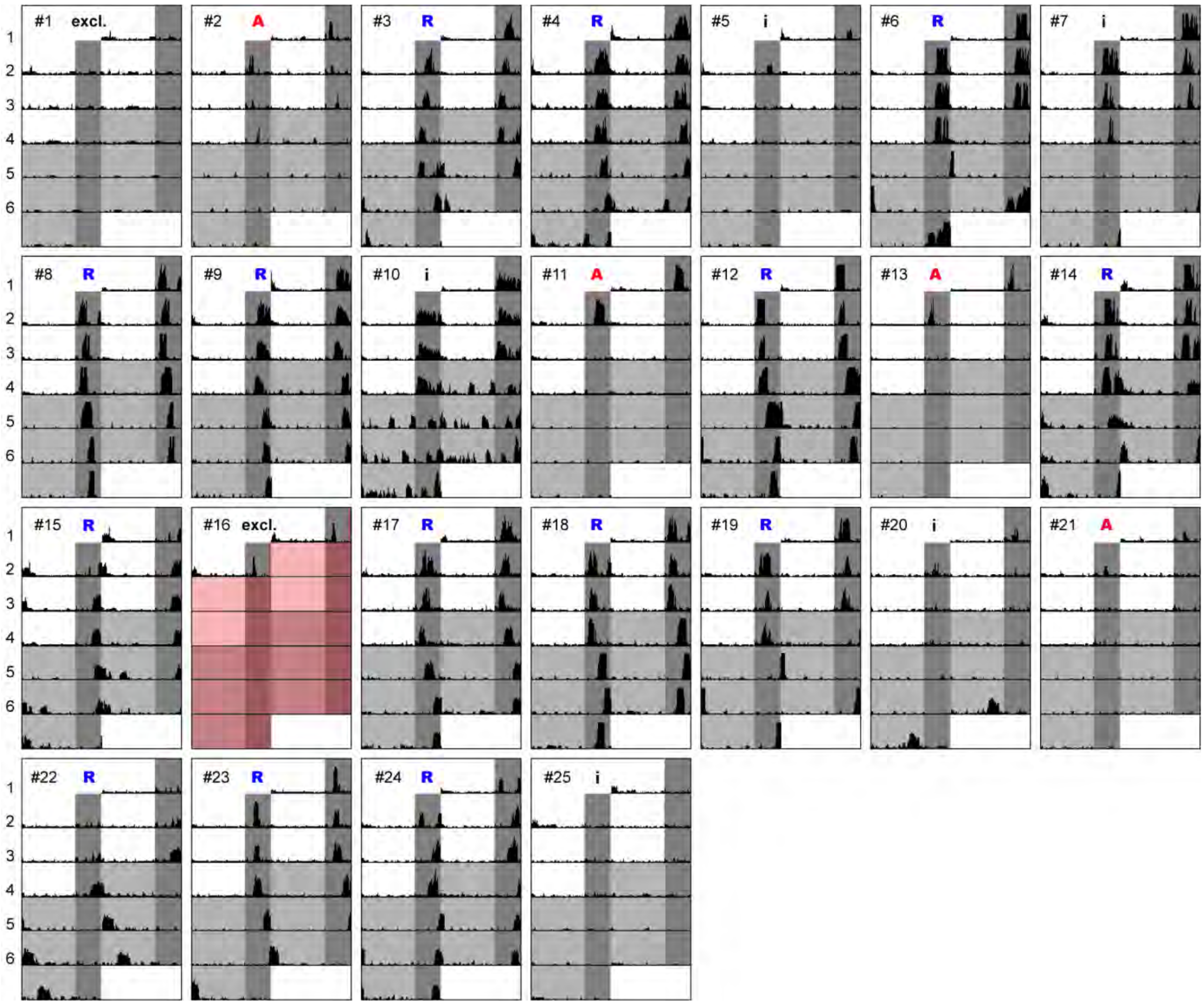

recording #3 (20.11.2019-25.11.2019, chamber 1)

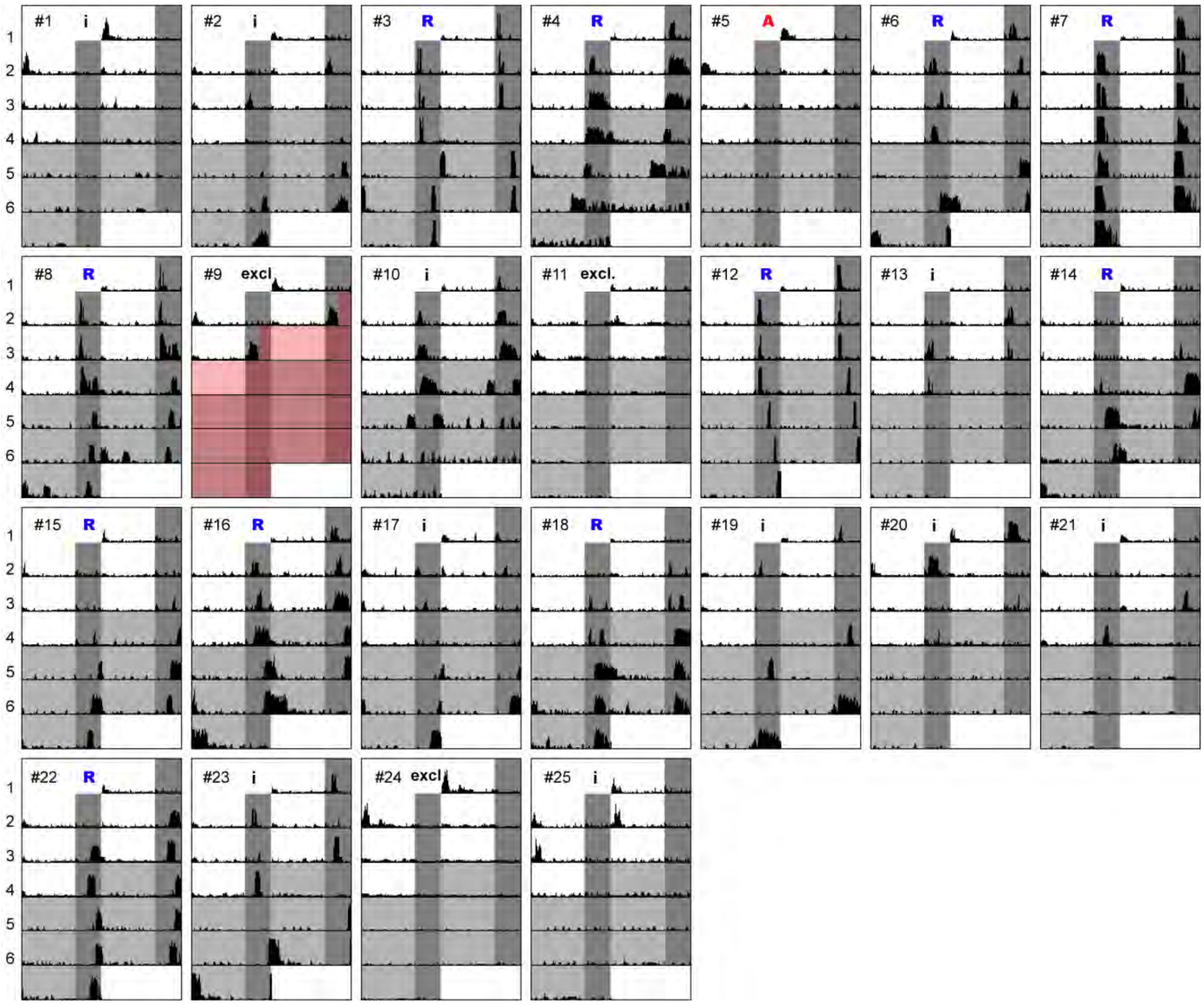

recording #4 (20.11.2019-25.11.2019, chamber 2)

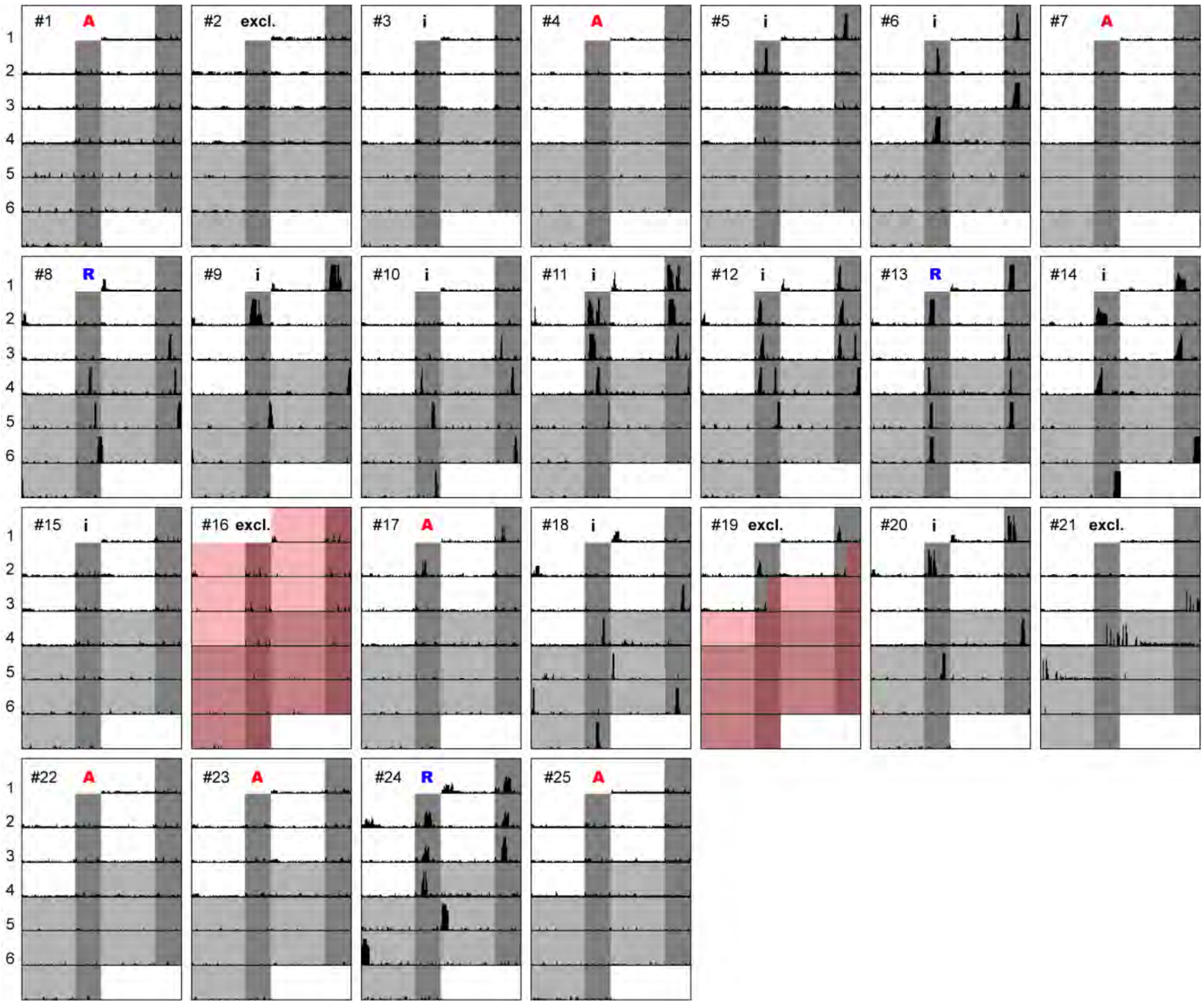

recording #5 (26.01.20-31.01.2020, chamber 1)

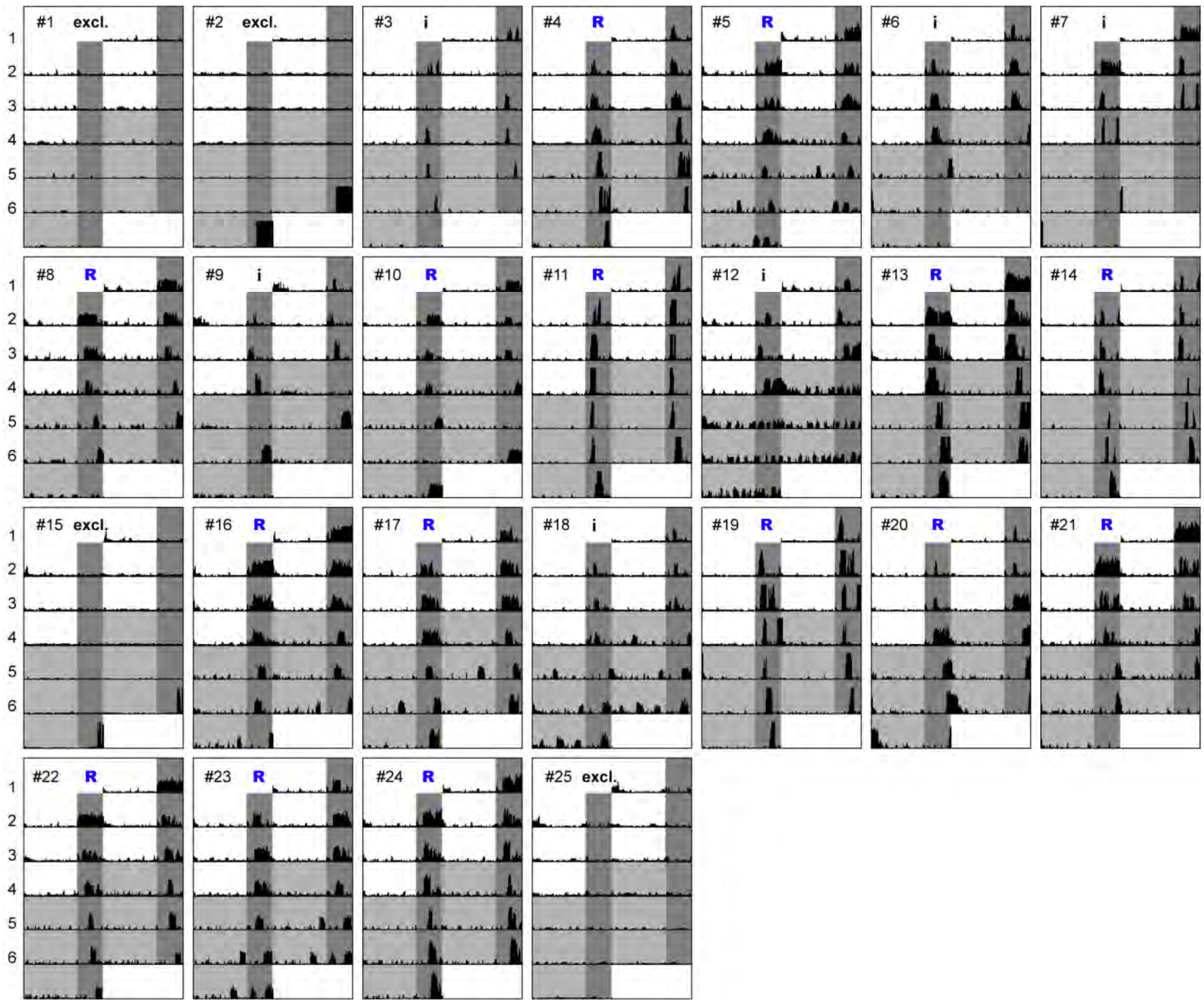

recording #6 (26.01.20-31.01.2020, chamber 2)

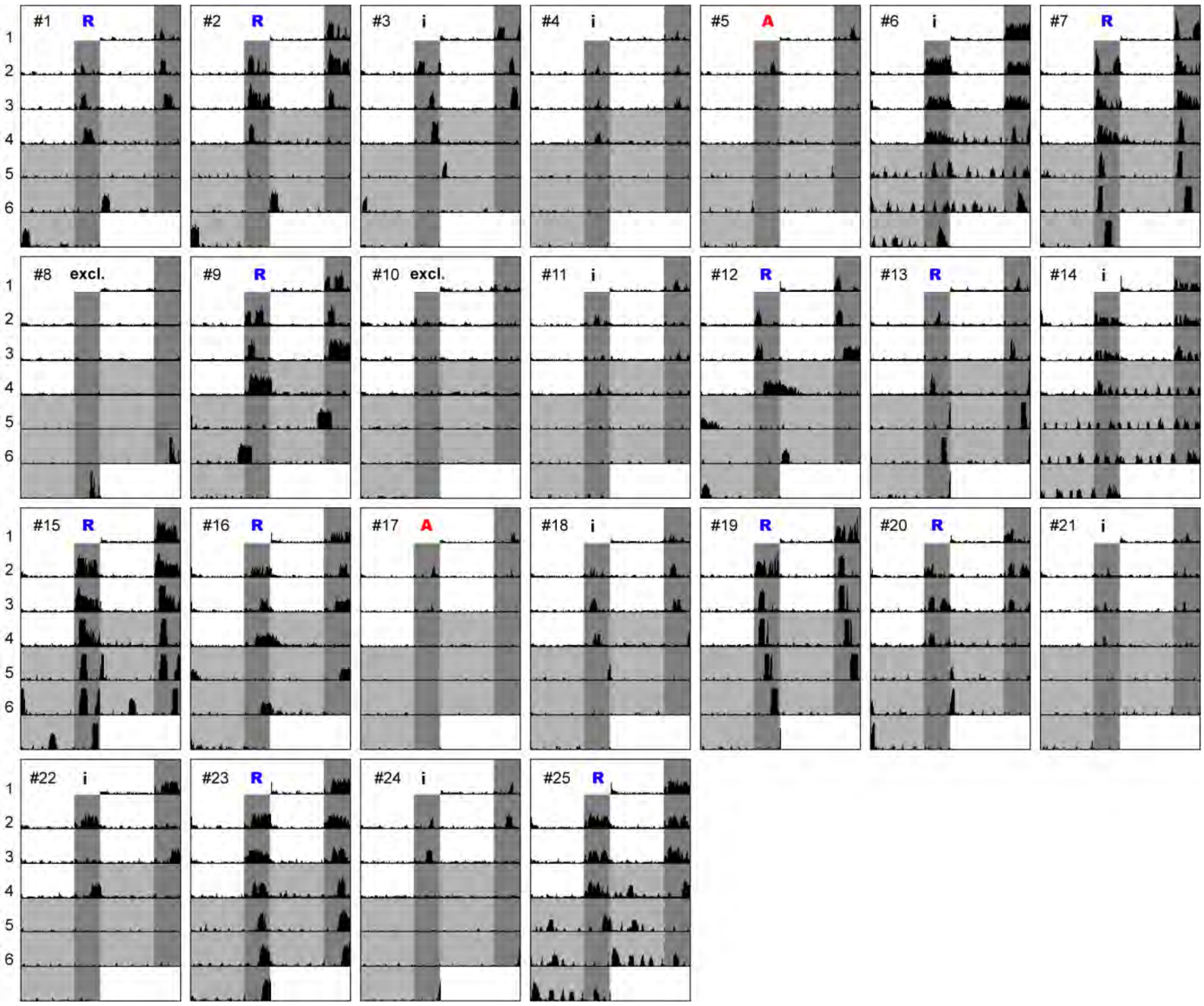

recording #7 (18.02.20-23.02.2020, chamber 1)

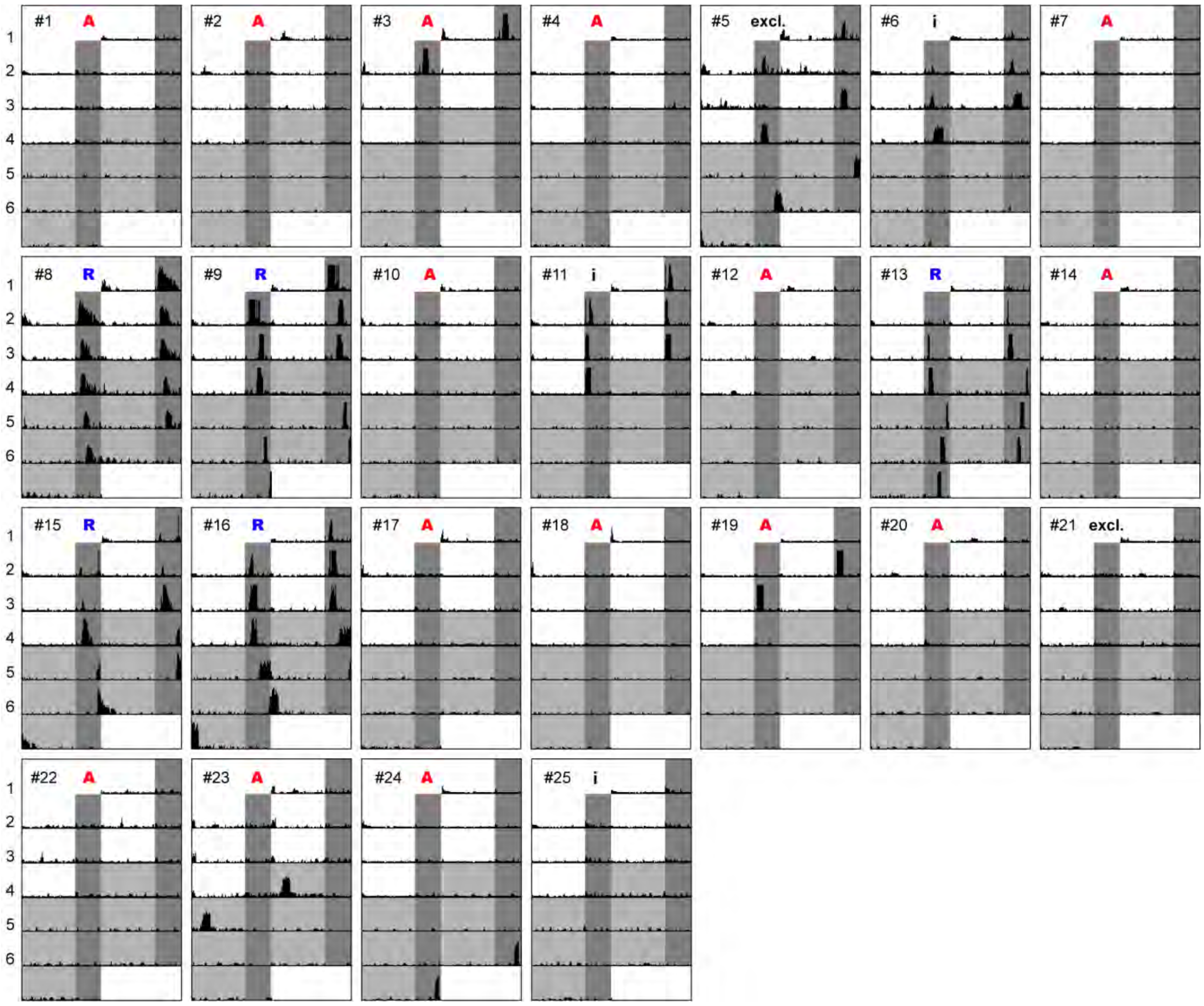

recording #8 (18.02.20-23.02.2020, chamber 2)

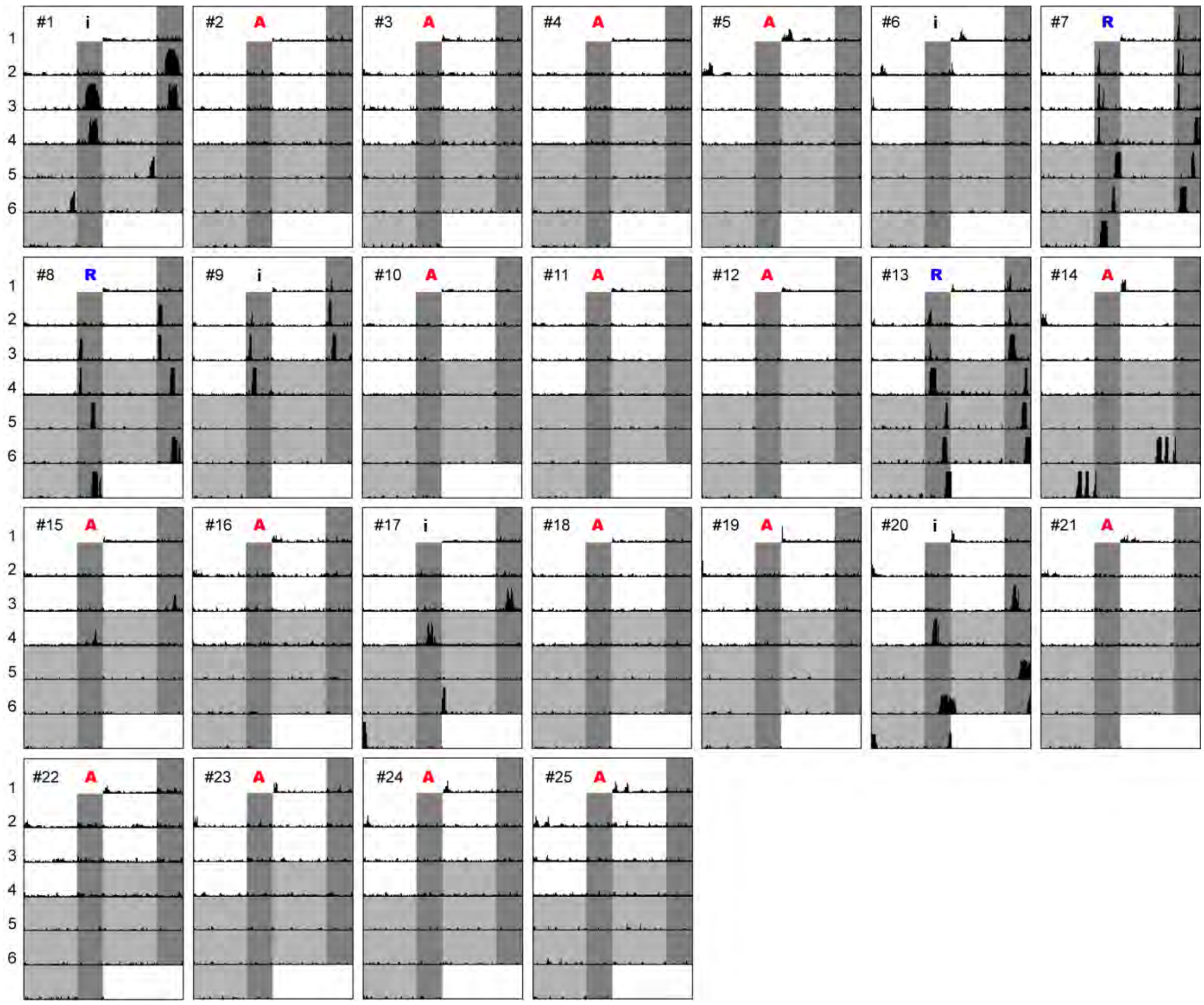

recording #9 (25.02.20-01.03.2020, chamber 1)

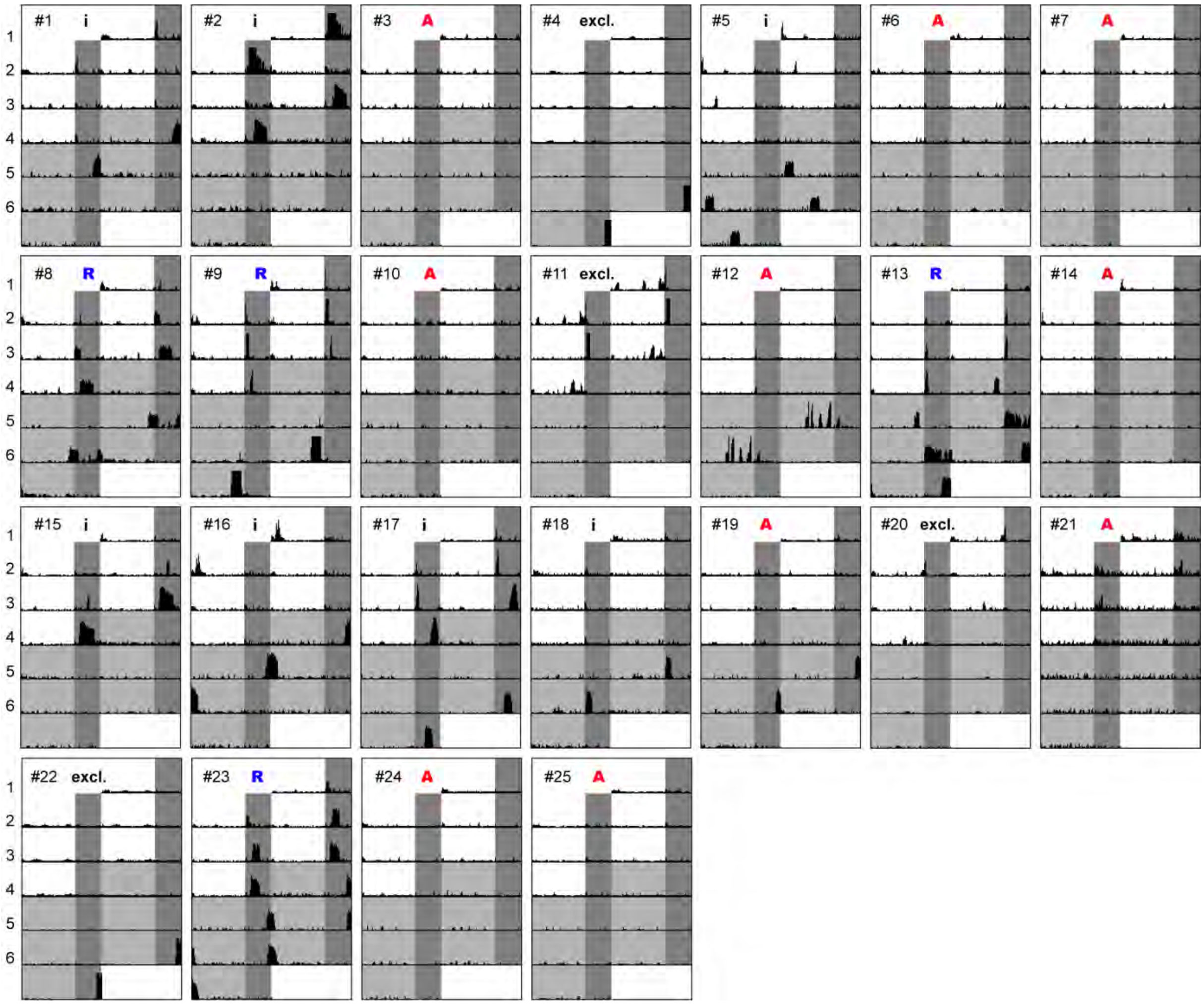

recording #10 (27.03.20-02.04.2020, chamber 1)

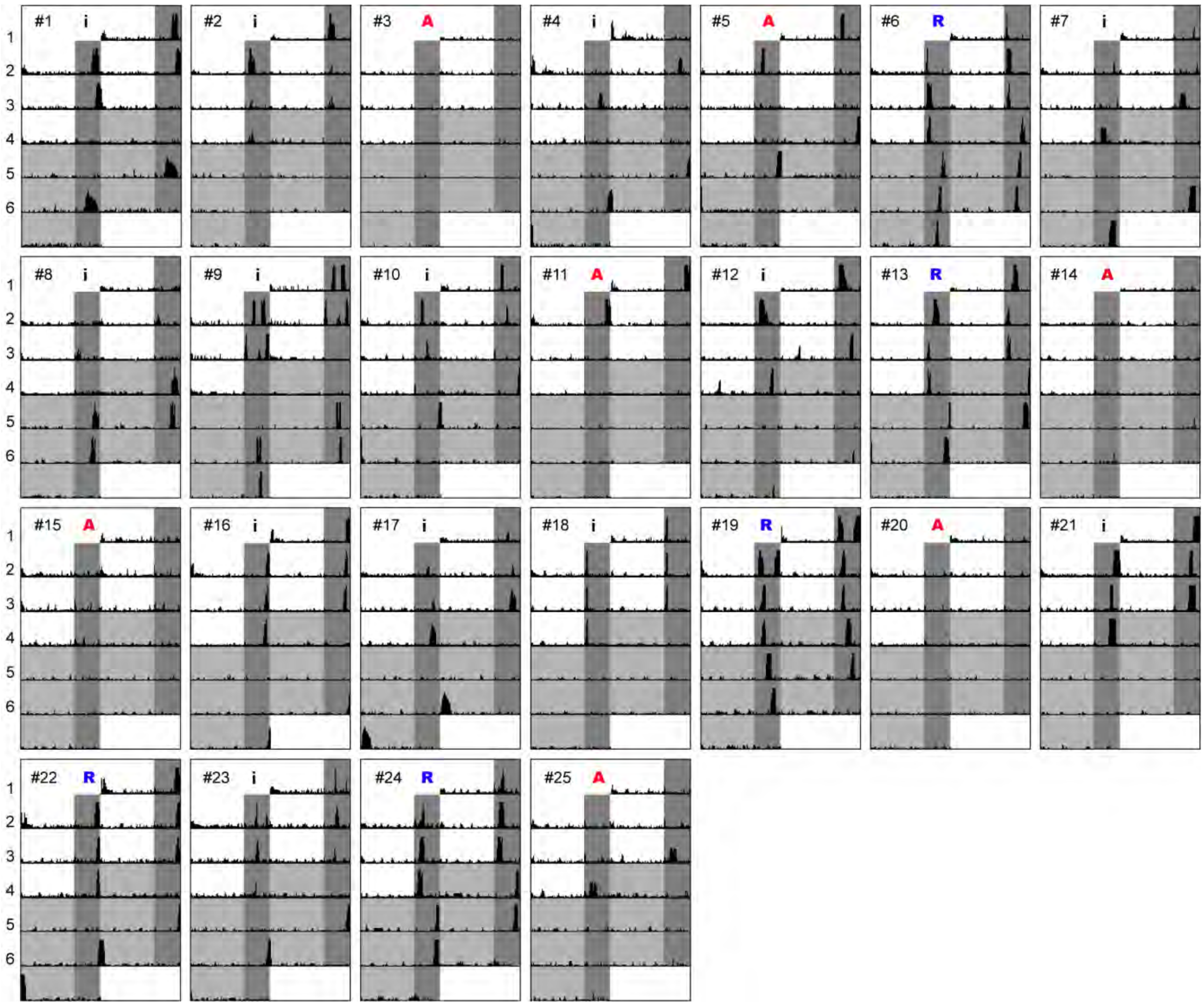

recording #11 (27.03.20-02.04.2020, chamber 2)

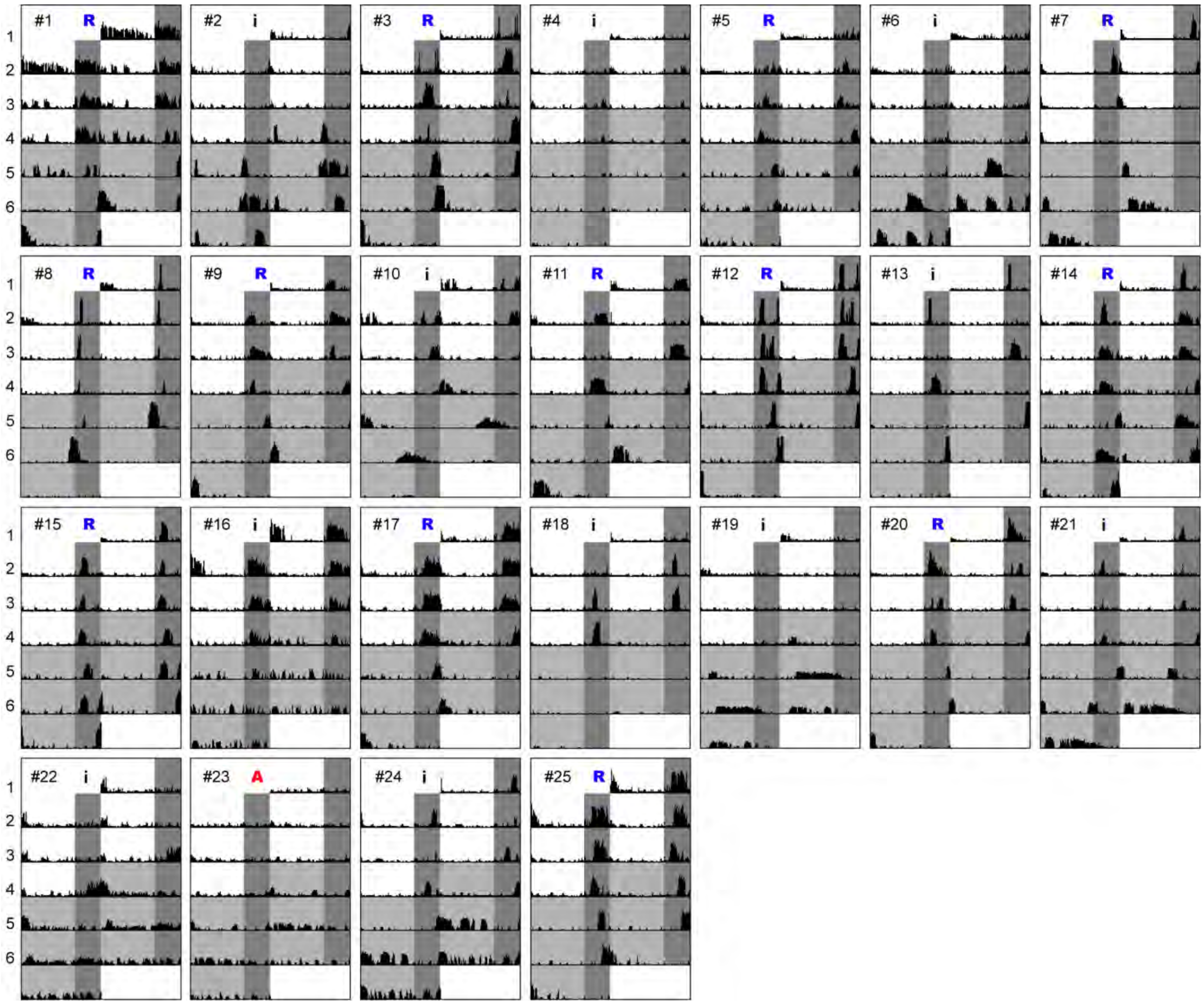

recording #12 (23.05.20-29.05.2020, chamber 1)

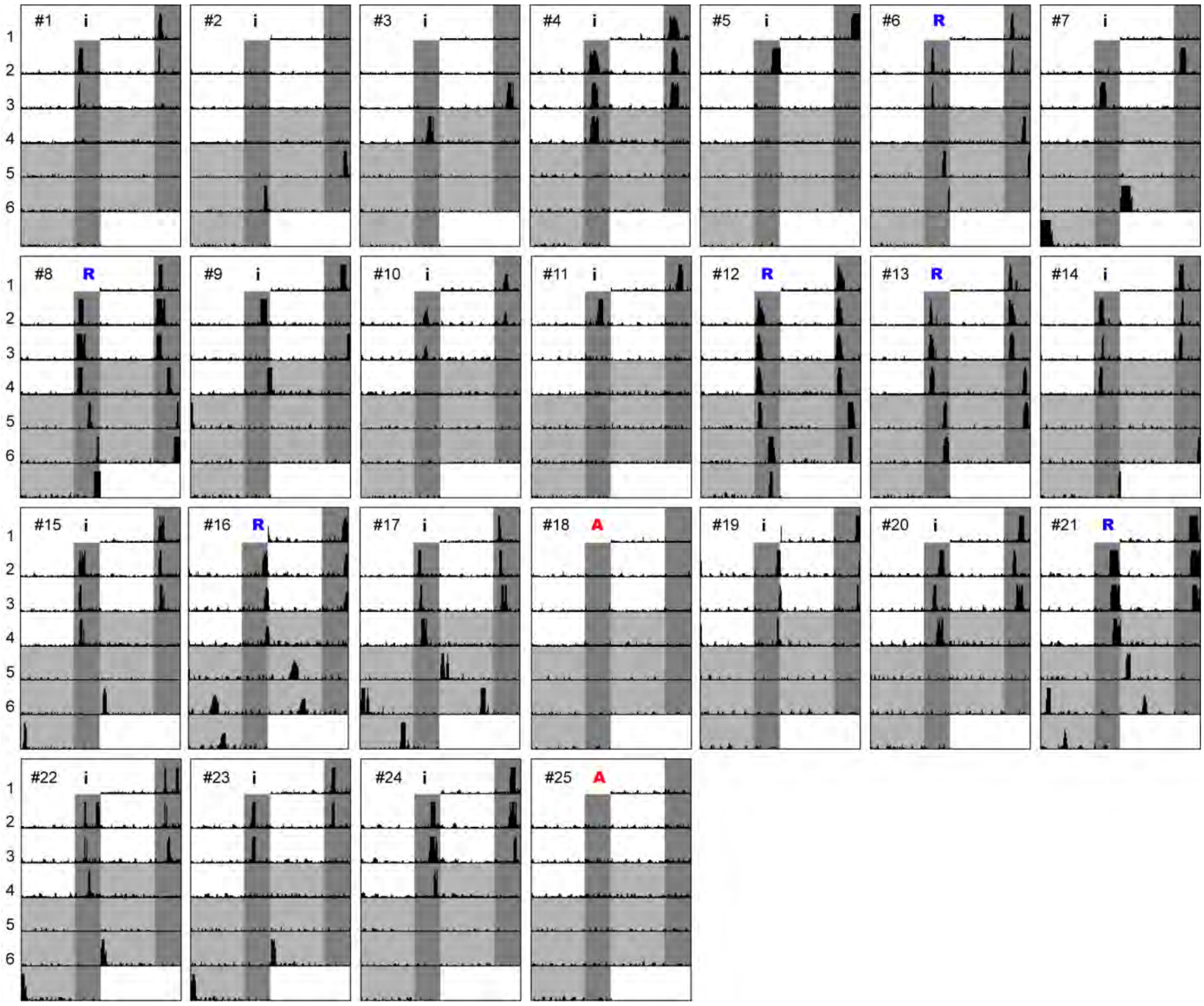

recording #13 (23.05.20-29.05.2020, chamber 2)

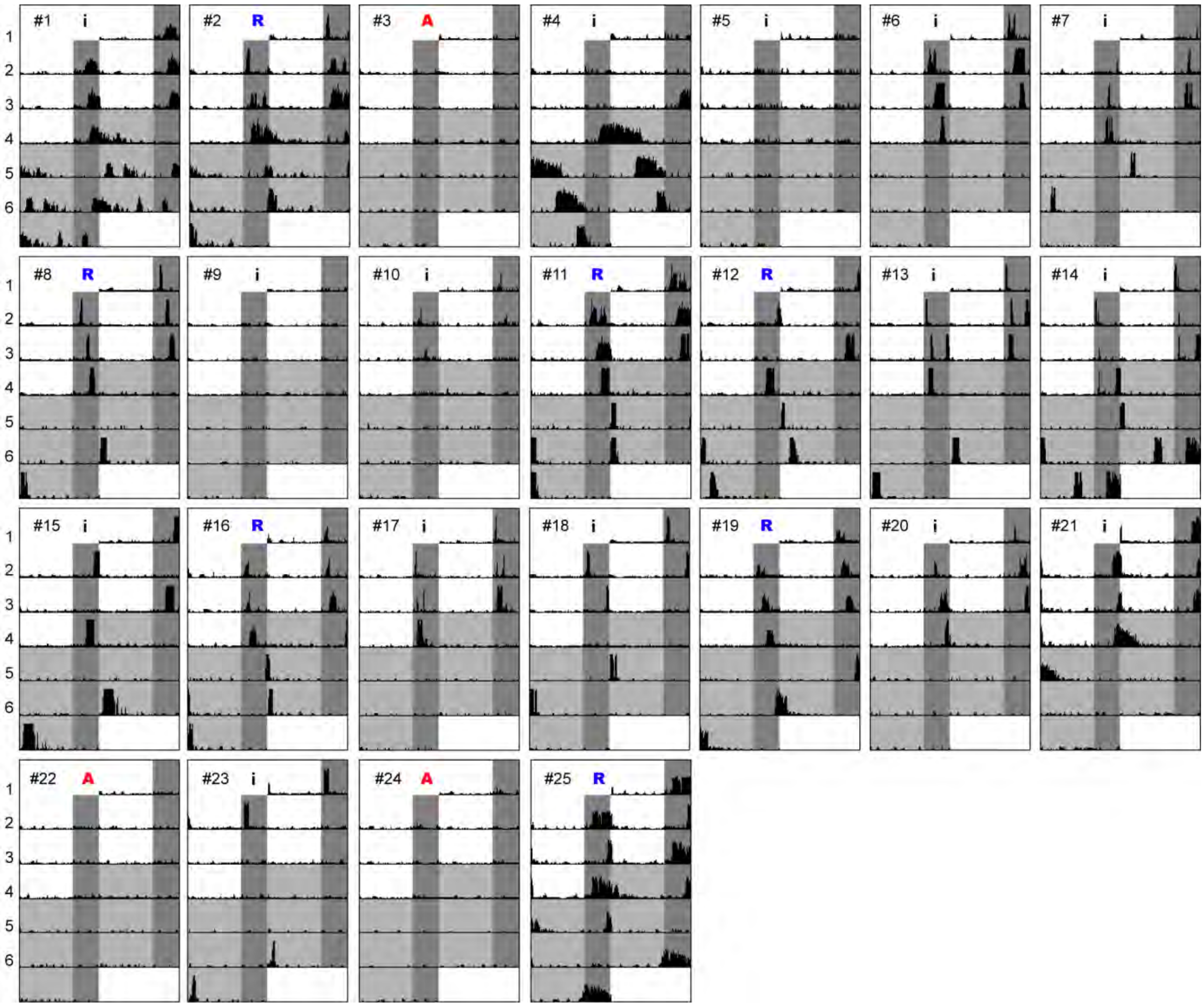

Supplement: S6 Fig — Related to Fig 2. Double-plotted actograms of individual PIN wild-type worms are shown. Locomotor activity was recorded over 3 d of LD (16 h:8 h) and 3 d of DD. Per behavioral recording, 25 worms were investigated in parallel. Two identical behavior chambers were used for recordings. Each page contains all worms of a characterization run including worms that matured and were thus excluded. #: individual worm identifier. Letters indicate characterization as rhythmic (R), arrhythmic (A), or intermediate (i). (PDF) [file pbio.3002572.s006.pdf]
