# Supplementary material for: Molecular circadian rhythms are robust in marine annelids lacking rhythmic behavior
Source: PLoS Biol. 2024 Apr 11;22(4):e3002572. doi: 10.1371/journal.pbio.3002572 (PMC11008795; doi:10.1371/journal.pbio.3002572)
Supplement: S7 Fig — Related to Figs 2 and S6. Double-plotted actograms of individual PIN wild-type worms are shown. Locomotor activity was recorded over 3 d of LD (16 h:8 h) and 3 d of DD. Y-axis is magnified 10-fold relative to S6 Fig to better illustrate activity patterns. (A) Worms characterized as arrhythmic. (B) Worms that crawled out and where no activity could be recorded. Red shading indicates that worms crawled out of the tracking well. For rec #4, worm #16, the animal left the tracking well before the start of recording but occasionally moved its head into the well, which was then tracked (panel C). This happened mostly when the worm was most active, i.e., during the dark phases. This comparison illustrates that although arrhythmic worms showed overall lower activity, they were far from inactive. Recording numbers and individual worm identifiers (#) match those in S6 Fig. (PDF) [file pbio.3002572.s007.pdf]

S7 Fig: Magnified individual actograms of arrhythmic worms for RNASeq analysis. Related to Fig 2 and S6 Fig. Double-plotted actograms of individual PIN wildtype worms are shown. Locomotor activity was recorded over 3 LD days (16h:8h) and 3 DD days. y-axis is magnified 10-fold relative to S6 Fig to better illustrate activity patterns. (A) Worms characterized as arrhythmic. (B) Worms that crawled out and where no activity could be recorded. Red shading indicates that worms crawled out of the tracking well. For rec #4, worm #16, the animal left the tracking arena before the start of recording, but occasionally moved its head into the well, which was then tracked (panel C). This happened mostly when the worms was most active, i.e. during the dark phases. This comparison illustrates that arrhythmic worms, though lacking distinct nocturnal activity bouts, were far from inactive. Recording numbers and individual worm identifiers (#) match those in S6 Fig.

A

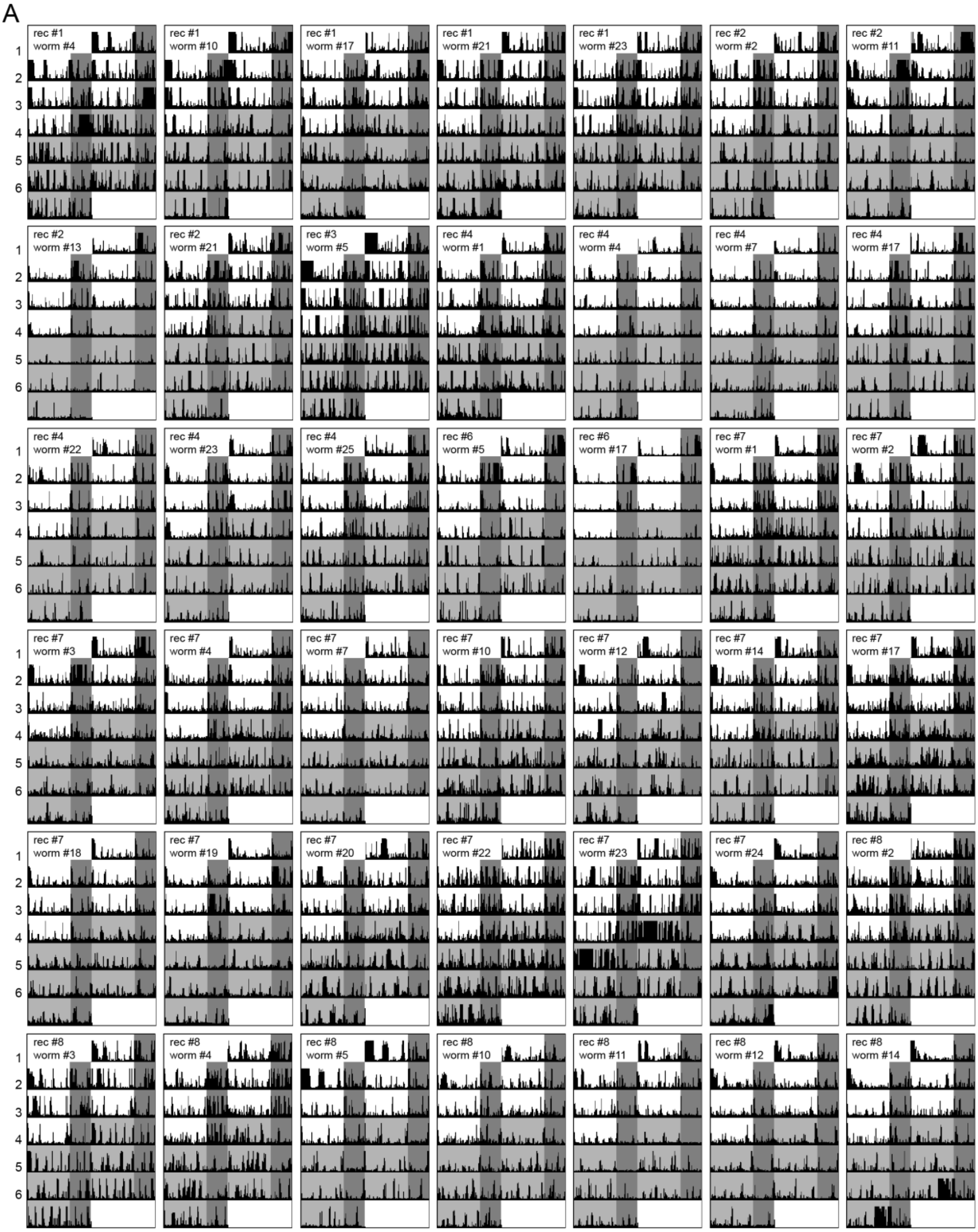

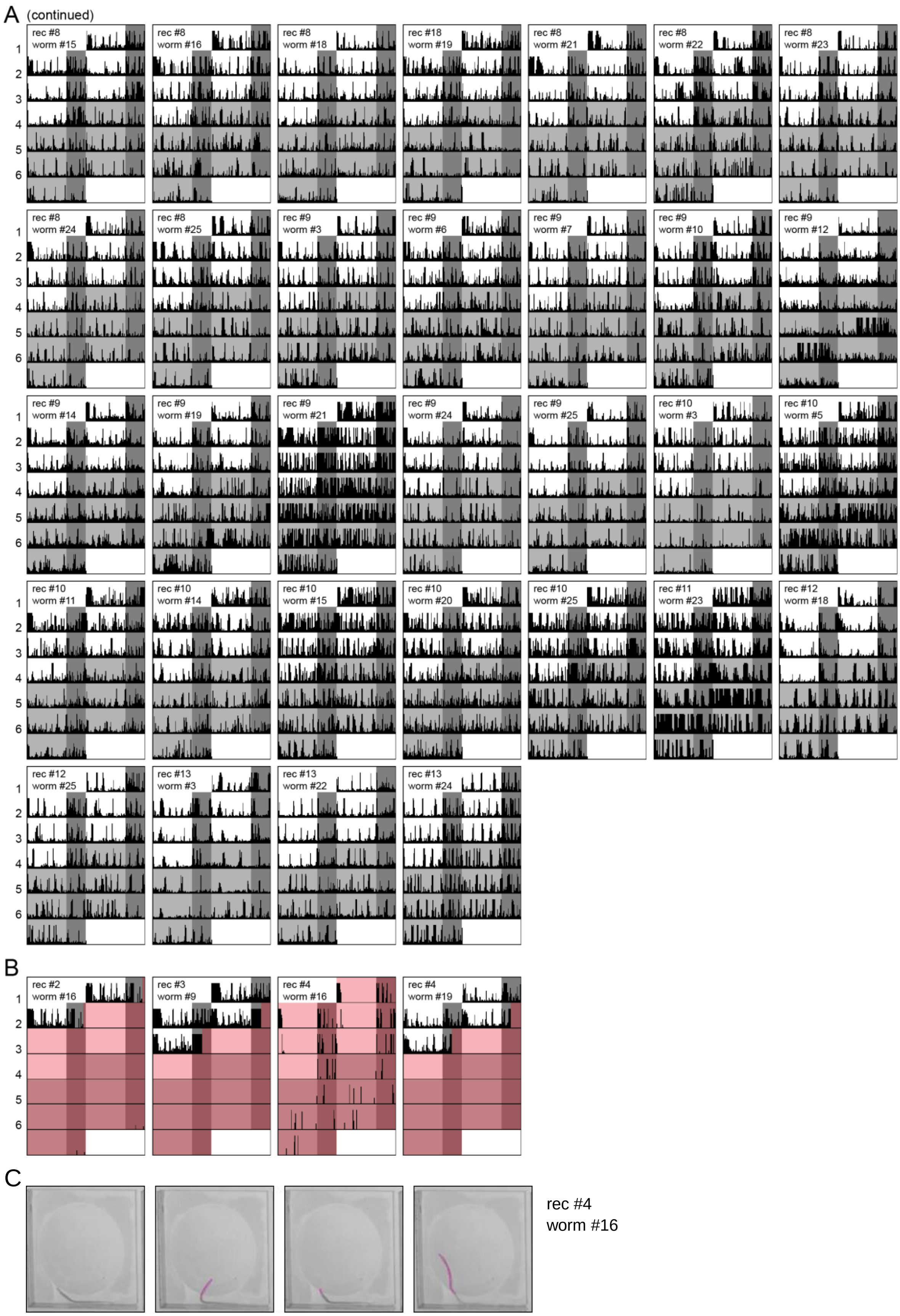
A (continued)
rec #8 worm #15
rec #8 worm #16
rec #8 worm #18
rec #18 worm #19
rec #8 worm #21
rec #8 worm #22
rec #8 worm #23
rec #8 worm #24
rec #8 worm #25
rec #9 worm #3
rec #9 worm #6
rec #9 worm #7
rec #9 worm #10
rec #9 worm #12
rec #9 worm #14
rec #9 worm #19
rec #9 worm #21
rec #9 worm #24
rec #9 worm #25
rec #10 worm #3
rec #10 worm #5
rec #10 worm #11
rec #10 worm #14
rec #10 worm #15
rec #10 worm #20
rec #10 worm #25
rec #11 worm #23
rec #12 worm #18
rec #12 worm #25
rec #13 worm #3
rec #13 worm #22
rec #13 worm #24
1
2
3
4
5
6
B
rec #2 worm #16
rec #3 worm #9
rec #4 worm #16
rec #4 worm #19
C
rec #4
worm #16
