# Supplementary material for: Molecular circadian rhythms are robust in marine annelids lacking rhythmic behavior
Source: PLoS Biol. 2024 Apr 11;22(4):e3002572. doi: 10.1371/journal.pbio.3002572 (PMC11008795; doi:10.1371/journal.pbio.3002572)
Supplement: S10 Fig — Shown are double-plotted actograms of mixed VIO/PIN background pdf wild types (A) and mutants (B) related to Fig 6, the initial VIO background wild types (C) and mutants (D) related to S11 Fig, and VIO backcrossed wild types (E) and mutants (F) related to S12 Fig. Locomotor activity was recorded over 4 d of LD (16 h:8 h) and 4, 5, or 8 d of DD. #: individual worm identifier. Genotypes of pdf mutants (−14/−14, +4/+4, −14/+4) are indicated at the bottom of the respective actograms. Red shading indicates that worms crawled out of the tracking well. Worms that were excluded from statistics due to maturation during or within 1 week after the recording are not shown, as maturation strongly alters their overall behavior. (PDF) [file pbio.3002572.s010.pdf]

<u>S10 Fig: Individual actograms of *pdf* wildtype/mutant worm comparison.</u> Double-plotted actograms of mixed VIO/PIN background *pdf* wt (A) and mutants (B) related to Fig 6, the initial VIO background wt (C) and mutants (D) related to S11Fig, and VIO backcrossed wildtypes (E) and mutants (F) related to S12Fig. Period/power values in S1D,E Table. Locomotor activity was recorded over 4 LD days (16h:8h) and 4,5 or 8 DD days. #: individual worm identifier. Genotypes of *pdf* mutants (-14/-14, +4/+4, -14/+4) are indicated at the bottom of the respective actograms. Red shading indicates when worms crawled out of the tracking well. Worms excluded from analyses due to maturation during or within one week after the recording are not shown, as maturation strongly alters behavior.

A

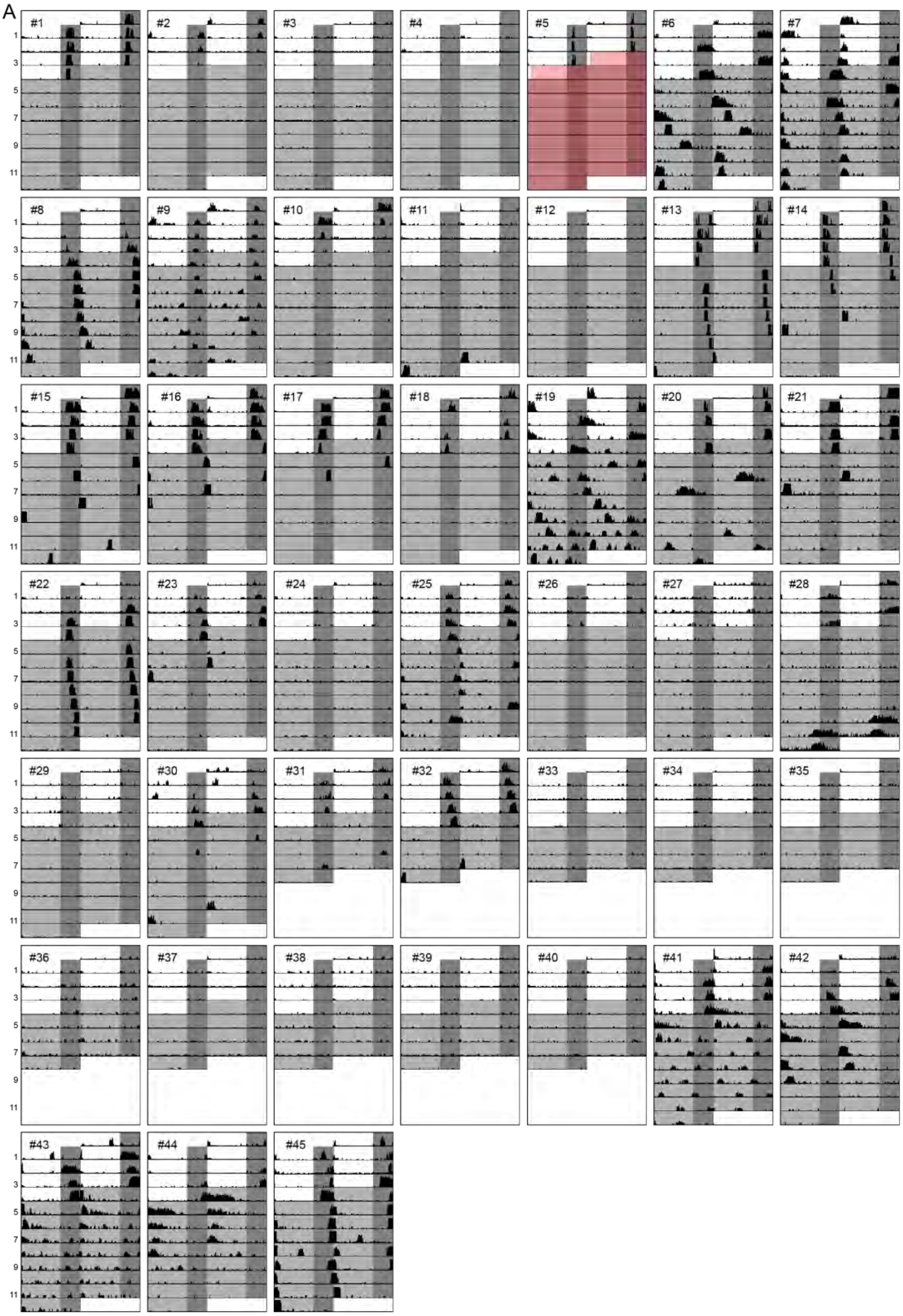

B

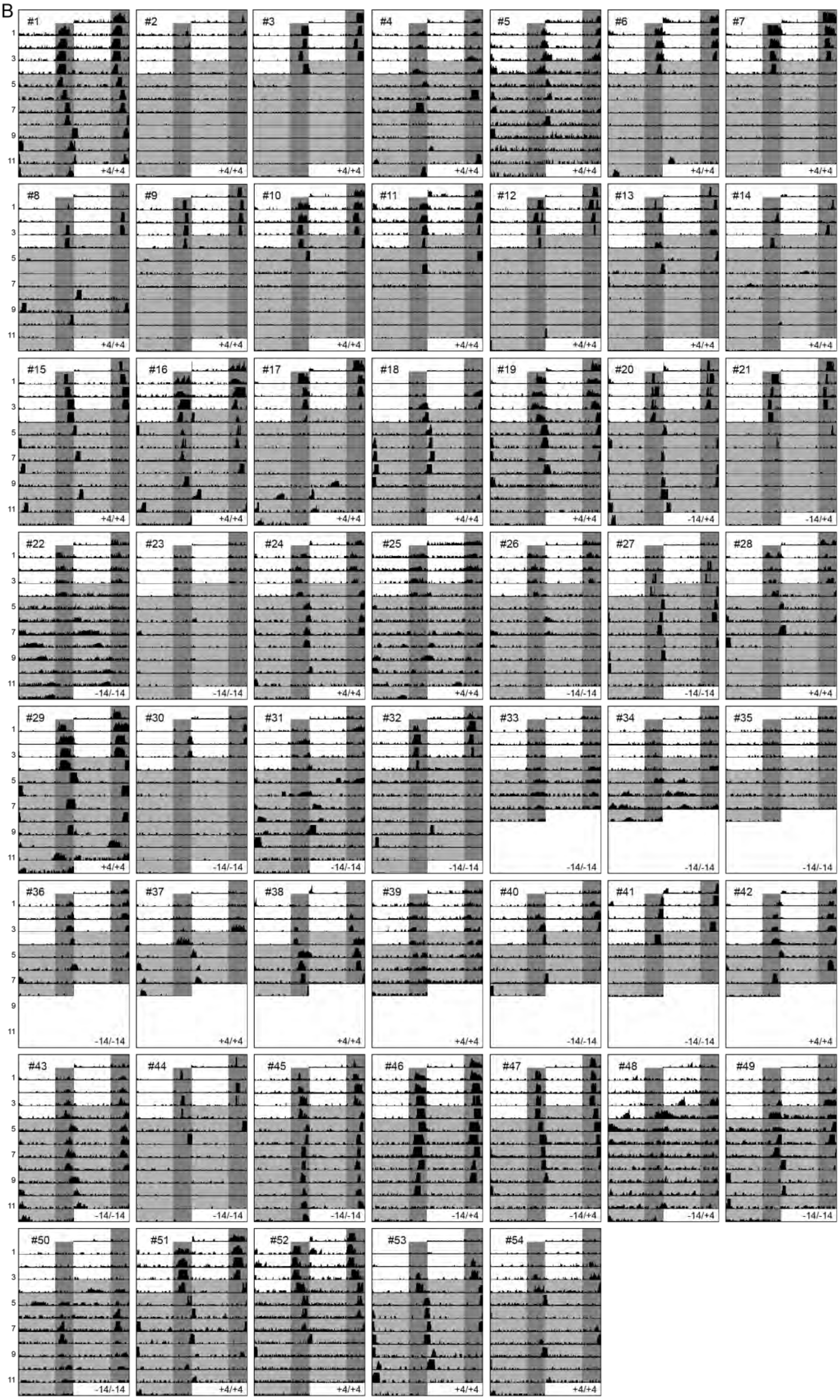

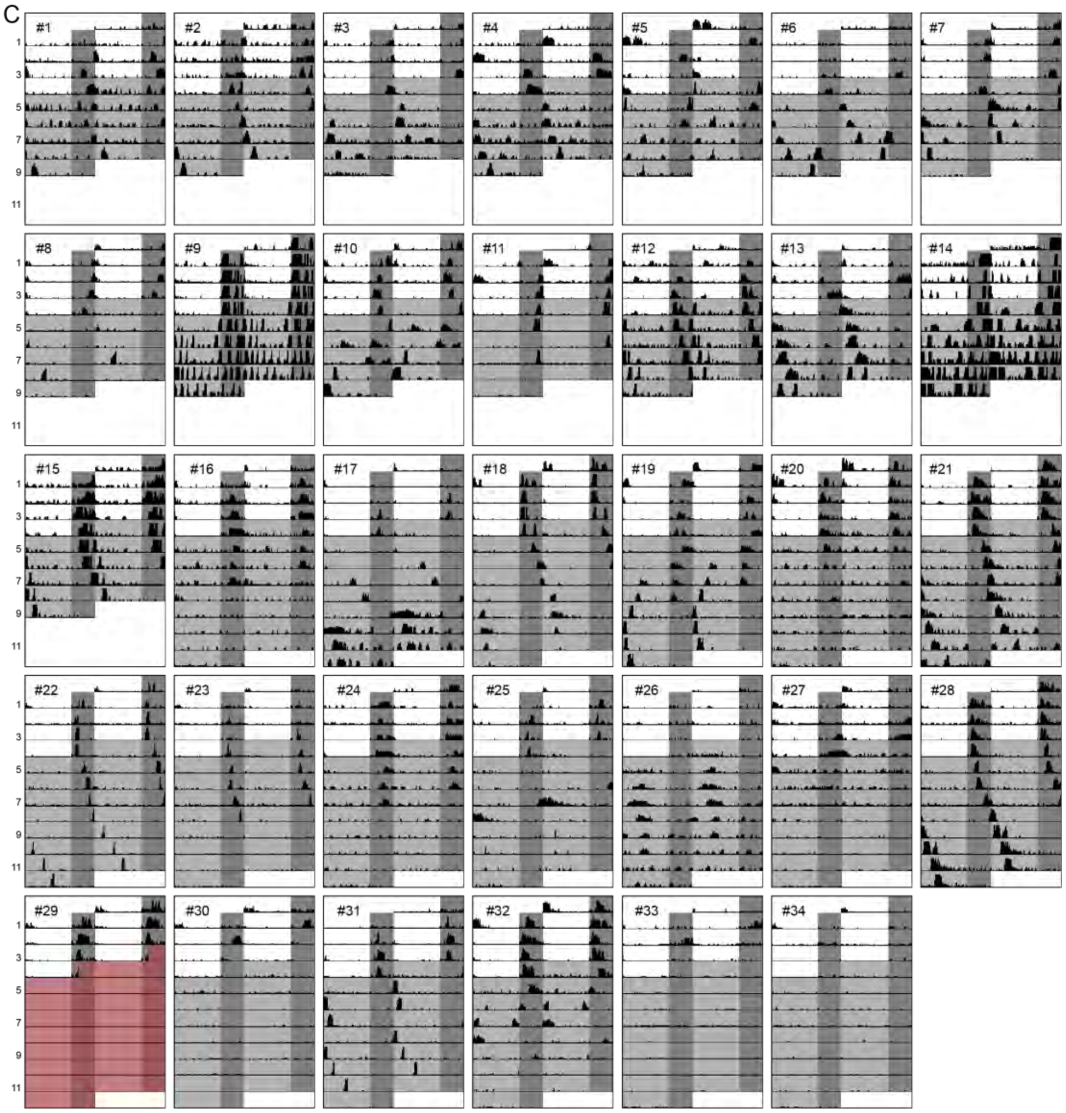
C
#1
#2
#3
#4
#5
#6
#7
#8
#9
#10
#11
#12
#13
#14
#15
#16
#17
#18
#19
#20
#21
#22
#23
#24
#25
#26
#27
#28
#29
#30
#31
#32
#33
#34

D

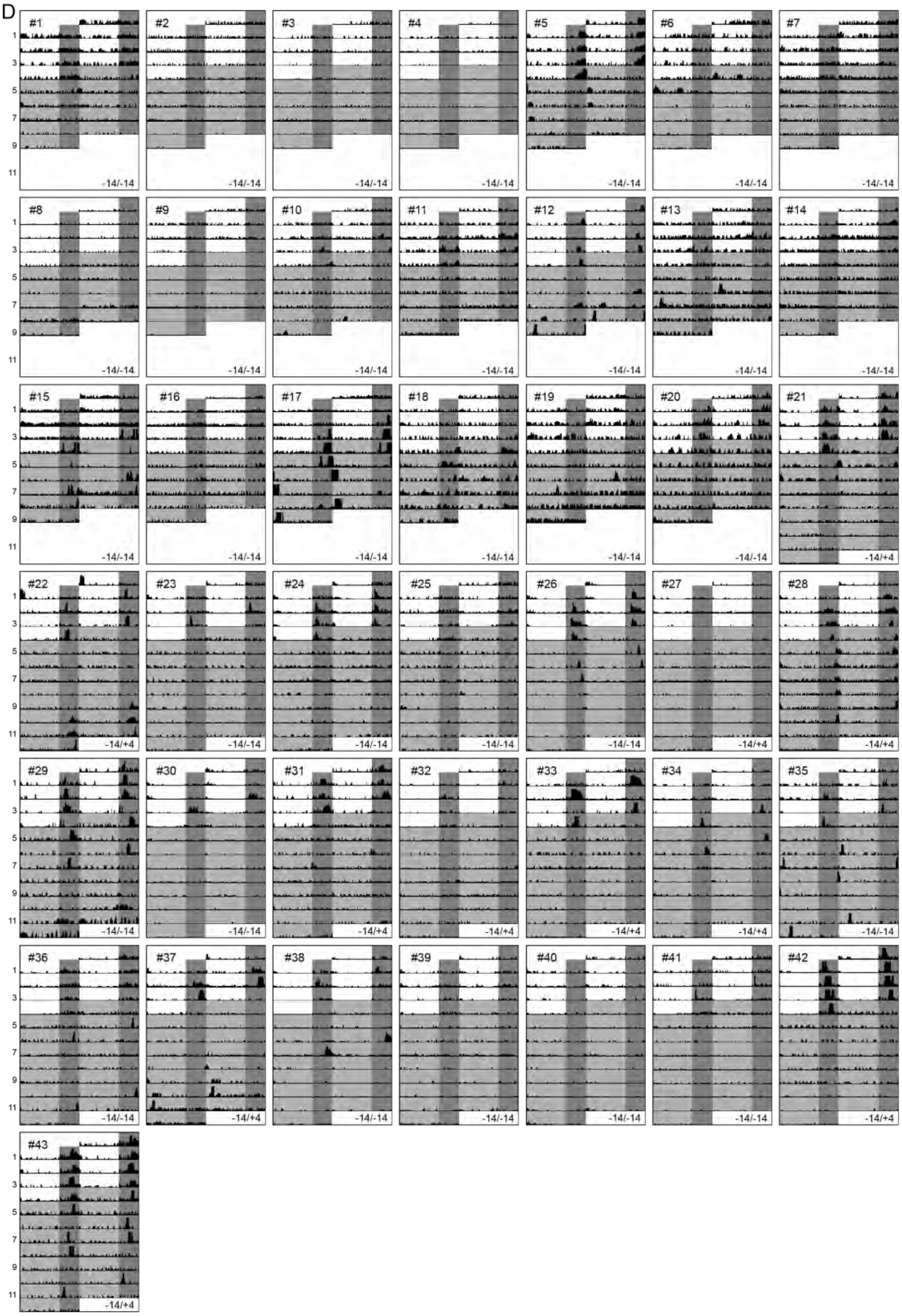

E

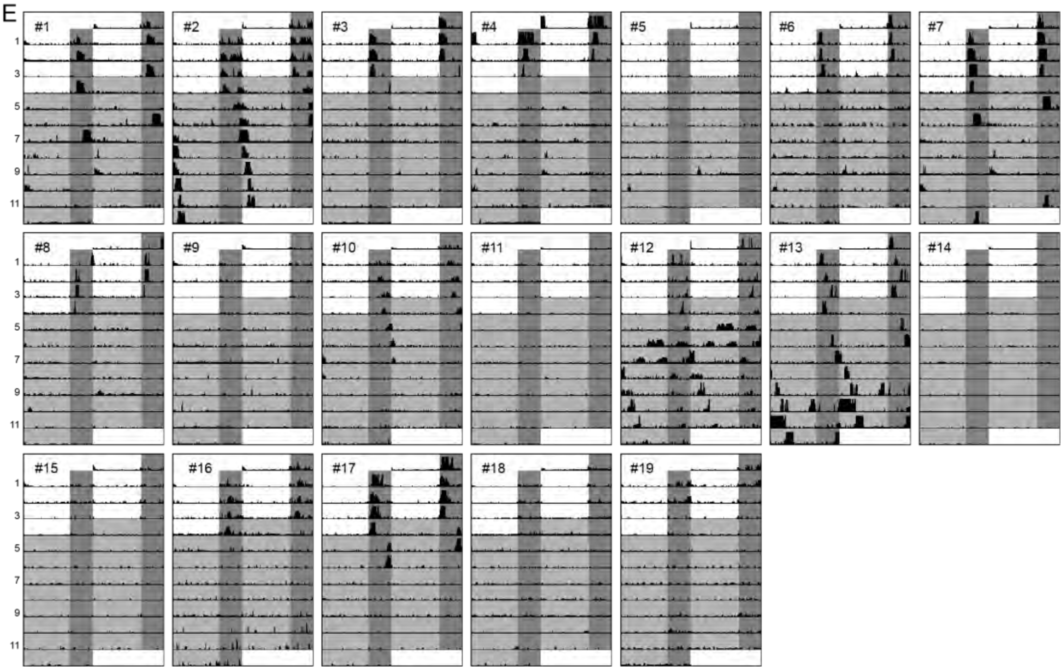
#1
#2
#3
#4
#5
#6
#7
#8
#9
#10
#11
#12
#13
#14
#15
#16
#17
#18
#19
1
3
5
7
9
11

F

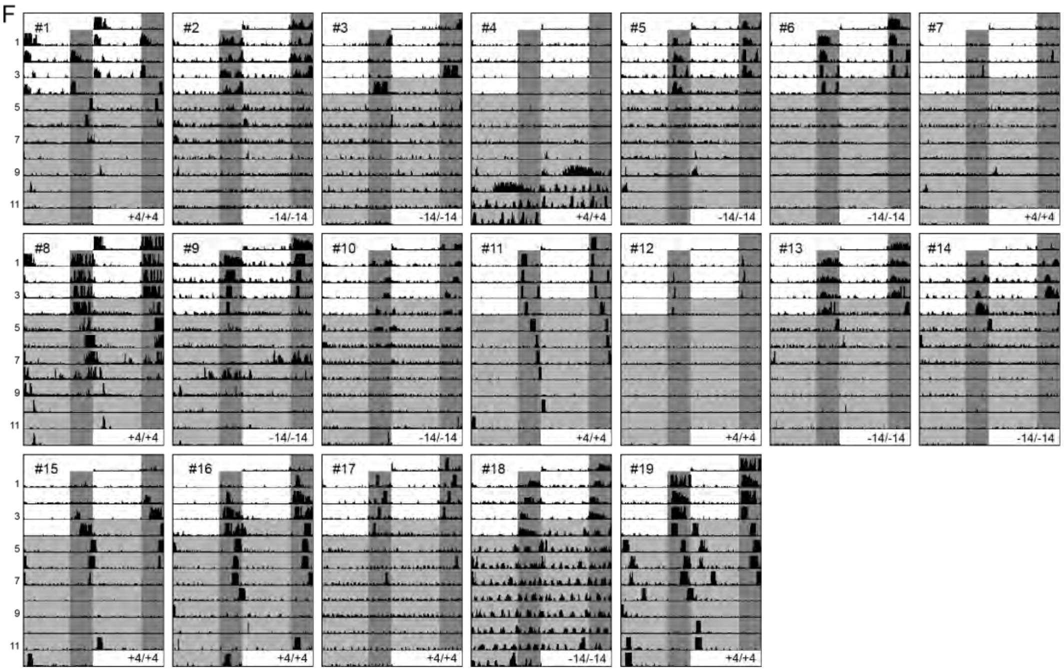
#1
+4/+4
#2
-14/-14
#3
-14/-14
#4
+4/+4
#5
-14/-14
#6
-14/-14
#7
+4/+4
#8
+4/+4
#9
-14/-14
#10
-14/-14
#11
+4/+4
#12
+4/+4
#13
-14/-14
#14
-14/-14
#15
+4/+4
#16
+4/+4
#17
+4/+4
#18
-14/-14
#19
+4/+4
1
3
5
7
9
11
