# Supplementary material for: Molecular circadian rhythms are robust in marine annelids lacking rhythmic behavior
Source: PLoS Biol. 2024 Apr 11;22(4):e3002572. doi: 10.1371/journal.pbio.3002572 (PMC11008795; doi:10.1371/journal.pbio.3002572)
Supplement: Attachment — Submitted filename: r-p_PlosBio_responses to reviewers_6.docx [file pbio.3002572.s020.docx]

## **Response to reviewers**

*We like to note that while the comments of the reviewers were extensive and required us to put substantial additional effort and time into our manuscript, they have clearly helped us to improve it significantly. We know ourselves how much time a careful and detailed review requires and are thus truly grateful to our reviewers for putting in this time, even though it meant more work for us. We valued their time and considered each comment carefully, even if we in some cases have a different opinion. But even in these cases it helped us to see how our wording might be misunderstood and helped to clarify the manuscript.*

*One general note concerning a piece of supplemental information: For file size reasons we were advised to upload S2 Table separately. Thus, S2 Table is available under: https://www.dropbox.com/scl/fi/uw3oiqaeh12gh3aksbtbn/Haefker-et-al_S2-Tab_final.xlsx?rlkey=wogylkz06z5q3ceczbmw1szrd&dl=0).*

**Reviewer #1**:

The manuscript "Behavioral rhythms are bad predictors of general organismal rhythmicity" deals with important questions related to the variability of behavior and gene expression circadian rhythms among strains and individuals of the marine annelid *Platynereis dumerilii*.

Circadian rhythms have the important function of synchronizing cellular processes to the diel periodicities and are crucial for the adaptation of organisms' physiology and behavior to the environment. Numerous studies have highlighted the fundamental role of these adaptation systems for organismal health and fitness, mainly in terrestrial organisms. One current challenge for the research community is to explore the role of these mechanisms in the marine environment, especially in the light of climate change and increasing photo-pollution. Thus the questions addressed in the manuscript are relevant.

The authors analyze the link between rhythmicity of behavior and cycling of transcripts over the 24 hour light-dark cycle. In particular, they produce actograms on individual worms from different strains reporting the locomotor activity and found strong variability in the behavior of different strains-individuals (some showing rhythms of activity and some being arrhythmic). They then performed transcriptomic analysis on the different samples and highlighted that despite this difference in behavior, all samples were showing rhythms in the transcription on circadian clock genes. In addition, the number of cycling genes was comparable between samples but the categories of cycling transcripts differed dramatically: in the strains showing rhythms of activity an enrichment of genes encoding for components of neuronal signaling was found while genes related to metabolic processes were the most cycling in arrhythmic worms. The authors speculate that this could be an adaptive
strategy to cope with different environmental conditions, increasing population fitness. Also, they point to the fact that using only behavioral tests in circadian experiments to assess organismal rhythmicity could be misleading.

Finally, using knockout lines they functionally analyse the potential role of Pdf, a neuropeptide known in Drosophila for its role in the control of locomotor activity rhythms, and discover that this function is not conserved in annelids.

In general, I found the manuscript very well written and technically sound. Overall the interpretation of data and conclusions drawn by the authors are reasonable and supported by the data and the manuscript contains new relevant information that will be of interest for a broad audience. I particularly appreciated the clarity of the figures, the depth of the analyses, the amount of data used to support the conclusions and the way in which the study was put in the context. I also believe this study will be of inspiration for other researchers working on similar questions.

*We thank the reviewer for her detailed and correct summary of our work and overall positive judgement.*

It will be important and extremely interesting to understand what mechanism is controlling the tradeoff between neuronal/behavioral transcriptional rhythms versus physiology/metabolism rhythms and if there is a sort of imprinting mechanism occurring at one phase of the worm life cycle. It is indeed puzzling that animals reared in identical conditions display such a level of variability in terms of circadian rhythms. As the authors suggest, stable strain specific epigenetic modifications could have a role in this process. One limitation of the study lies in the fact that the authors are using strains that have been collected decades ago from the environment and since then have been reared in laboratory conditions. It will be interesting to explore the discovered behavioral and transcriptomic variability in natural populations.

For all the above mentioned reasons, I consider the manuscript worthy of publication in Plos Biology.

*Again we thank for the positive assessment. We agree that the question concerning the variance in natural populations is a very interesting one. However, we decided to first analyze behavioral variance in the lab incrossed populations and non-incrossed, but closed-culture strain, as in many cases the full scale natural variance can produce results that make it difficult to deduce principle rules. Also, it should be noted that all existing alleles in our culture are reflecting natural alleles and are thus also relevant. The analyses of genetically highly incrossed lines with minimized genetic diversity allowed us to show that the observed reproducible individual diversity is present even in closely related worms.*

*Having established the principle phenomenon, we will be very excited to then generate a follow up study which studies these behavioral and molecular phenomena in the more diverse natural population.*

**Reviewer #2**:

This is an interesting study that aims to elucidate the relationship between individual-level behavioral rhythmicity (or lack thereof) and organismal transcriptional rhythms. This is an important question, given the widespread use of behavioral rhythmicity as an indicator of circadian clock function in many animal models.  The study is also significant as inter-individual variability in expressing behavioral or physiological rhythms could be a potential mechanism for species adaptability to changing environmental conditions.

The authors used the sea worm *Platynereis* for their study.  They studied variability of circadian behavior between different strains of *Platynereis* and between individuals. An interesting idea was to determine whether individuals behave similarly when tested on two separate occasions, and the authors claim that this is usually the case, thus suggesting that individuals express reproducible rhythmic "personalities".  The authors then selected behaviorally rhythmic and arrhythmic animals, and measured at the transcriptome level rhythmic gene expression under LD (light/dark) cycles. Correlates between behavioral rhythmic phenotypes and transcriptomic rhythms are proposed to be present.  This would be a very interesting observation. The authors end their study by showing that PDF is not required for behavioral rhythms in *Platynereis*, while it plays an important role in insect behavioral rhythms. There are clearly innovative ideas in this large body of work, but it unfortunately
suffers from significant weaknesses that undermine the solidity of the main conclusions.

*We thank the reviewer for the pointing out that our study is in principle highly interesting work, with particular relevance for many animal studies that use behavior as proxy for circadian clock functions. As can be seen, we fully addressed all skepticism concerning the reproducibility of individual behavioral phenotype, as well as the relationships between behavioral and transcriptomic rhythms in the respective sections below. We hope that the reviewer now agrees that our conclusions are sufficiently supported by our data and (two independent statistical) analyses.*

Major concerns:

1.	The authors propose that under LD conditions, there are transcripts that exhibit rhythms (a) only in behaviorally rhythmic worms; (b) only in behaviorally arrhythmic worms, and (c) in both. Given this, it seems that behavioral rhythms are not bad predictors of organismal rhythmicity, but actually quite good predictors for an important subset of organismal rhythms. For this and two more reasons, I think the title needs to be changed.  Indeed, it sounds flippant ("bad" in particular), and it is too broad: it is not clear yet that what was observed in *Platynereis* will apply to other species.

*This is certainly a relevant point that also led to significant discussions within the group. Of note, this point was also raised by reviewer #3. We modified the title to:*

*“Behaviorally rhythmic and arrhythmic individuals of a marine annelid exhibit similar levels of molecular rhythmicity in the brain”*

*Concerning the potential general significance of our finding it should be noted, however, that in a recent study of sensory conflicting environmental cues, the behaviors of the sea anemone* Nematostella vectensis *became arrhythmic, but highly consistent with our study, this was not reflected on the transcript level. We have included this recent study in our discussion section (doi: 10.7554/eLife.81084), see lines 556-562: “Of note, a study under sensory temporal conflict conditions in the sea anemone* Nematostella vectensis…”

2.	There are significant concerns with the methods used.   For example, when performing repetition runs on individual worms, the authors state that "actograms of the first and second recordings were very reproducible showing the same behavioral characteristics in both runs" (page 7, line 179). There are important issues here.  First, animals that appear arrhythmic seem to have very low activity.  Are they sick, or for any other reason simply not prone to movement? It seems, when looking at the individual actograms, that a large fraction of the intra-individual reproducibility comes from these inactive animals.

*As this aspect was raised by two reviewers, we apologize that this point was apparently not made sufficiently clear, but of course we would have excluded any even potentially sick animal from our analyses. For any of our experiments we do of course check the worms for their health. We have no indication that these worms were in any way less healthy than more active/rhythmic ones. All worms used for behavior experiments were visually investigated for signs of sickness (twitching, lack of tube-building, changed color, inclusions/ulcers) and were only used when there were no abnormalities. We now included this aspect in detail in the manuscript. (lines 641-643: “All worms were inspected for signs…”, lines 747-758: “To further ensure that the supposed inactivity of arrhythmic …”)*

*The confusion probably occurred, because we should have illustrated better that the arrhythmic worms are actually not inactive, but merely lack a nocturnal increase in locomotor activity. The existing lower, but not temporally “bundled” levels of activity are often not very well visible when the focus of the thresholds of the activity plots is on the activity bouts. To show baseline activity more clearly, high activity bouts would have to be cut off and then those would be neither properly quantifiable nor comparable across worms, which is why we usually set the thresholds in these activity diagrams the way we did.*

*To better illustrate this point, we added two supplemental figures (S4 Fig and S7 Fig) that re-plots all worms characterized as arrhythmic and seemingly lacking activity from Fig 1, S2 Fig* and *S7 Fig with a 10-fold scale increase of the y-axis.*

*For further illustration, in S7B,C Fig we also show actograms of worms that crawled out of the tracking wells to illustrate the stark difference between an arrhythmic worm that is moving regularly and background tracking noise.*

*Regarding the suggestions that the worms are “for any other reason not prone to movement”, it is again important to clarify that rhythmic and arrhythmic worms did not differ in their daytime activity (whether in LD or DD), but that arrhythmic worms did not show the nocturnal activity peak rhythmic worms did. The possible reasons for this are what we explored with our investigations of the clock genes and the transcriptome. Additionally, it is important to emphasize that arrhythmic worms were no outlier group, but part of a behavioral continuum with rhythmic and arrhythmic worms at the ends and various intermediate phenotypes in between.*

*Furthermore, it is not uncommon in circadian clock mutants in mice and Drosophila to have overall lower activity, e.g. bmal -/- mice*: [doi: 10.1371/journal.pone.0138661,](doi:%2010.1371/journal.pone.0138661,) *cwo -/- flies:* [*doi: 10.1177/0748730407313817*](doi:%2010.1177/0748730407313817). There is nothing “sick” about this phenotype in general.

*An additional point that argues against the investigated arrhythmic worms being in any way “unusual” or sick: In the group of worms characterized for RNAseq analysis and maintained for sampling, we saw that in the time between behavioral characterization and sampling (~1 month) maturations and spawnings occurred in both rhythmic and arrhythmic worms at the same lunar phase, over the same duration of the maturation period and with the same reproductive success. Swimming speed during their mating dances was indistinguishable. This aspect is now also mentioned in the manuscript: lines 754-758: “*Additionally, as some worms matured between…”

*In summary, there is no indication that behaviorally arrhythmic worms have a lower fitness than others and hence there is no reason to not consider or question these worms.*

3.	When looking at animals that actually showed strong behavioral activity in at least one of two runs, reproducibility is not obvious, at least to this reviewer.  Also, the authors simply tell us that behavior is reproducible within an individual, but there is no quantification, no criteria for similarity.  The authors should define quantifiable behavioral characteristics (activity level, period, rhythm power, phase) and use them to compare first and second recordings, and then show that the intra-individual correlation between the two runs is statistically stronger than if random pairs of animals were compared.

*As this point was also raised by reviewer #3, we put significant time investment into improving our analyses method to be more quantitative. We implemented two fully independent statistical approaches, which differ in the amount of complexity reduction of the data, to ensure that such reductions (which have to be done to some extent in any dataset) are not influencing the overall result. Both of our analyses show that repetition runs from the same, matching worms are significantly more similar in their behavioral profile, compared to the repetition runs of other, non-matching worms.*

*We first used a binary activity overlap approach, where the activity profile of each worm behavior run was transformed into a binary activity vector and then compared with the repeat run and the mean of all activity scores of all other repeat runs. The second approach is based on an already published statistical comparison method for locomotion similarity of behavioral patterns in the marine crab Uca (doi: 10.1371/journal.pone.0175403). It employs Kolmogorov-Smirnov tests to compare behavioral patterns between matching initial/repetition runs as well as to all other (non-matching repetition runs). The resulting p-values from each matching initial/repetition-comparison are treated as proxies of similarity and were compared to the remaining (non-matching) repetition runs via single-sample Wilcoxon signed-rank tests.*

*The results of the first, binary approach are now visualized in S5 Fig, while the results of the second approach were added to the initial/repetition actograms in Fig 1E and S3 Fig as scatter plots next to the respective actograms.*

*Importantly, both methods fully confirm our original statement of a higher similarity of repeat runs from the same individual than repeat runs from other individuals, especially for PIN strain worms. Both quantitative behavioral methods also support that behavioral rhythmicity vs. arrhythmicity is a non-random behavioral feature of individual worms. We believe that this forms as solid basis for our subsequent sampling for molecular analyses.*

*The description of the analysis was added in lines 212-243****:*** *“In addition to visual inspection of initial/repetition similarity, we…” and lines 699- 720: “Similarity analyses of individual worm behavioral patterns…”*

*Importantly, while we agree that quantitative behavioral approaches were missing and needed, we also want to point out the risk of reducing complex behavioral patterns by any quantitative method. Hence, in addition we also always ensured any conclusion by visual verifications.*

4.	The authors collected RNAseq samples from animals that have been identified as either behaviorally rhythmic or arrhythmic to assess how transcriptional rhythms differ in each cohort. However, there are several choices in designing this experiment that are of concern. First, the authors indicate that they mostly used DD for calling animals rhythmic or arrhythmic.

Then why did they do their transcriptomics studies under LD, under which many more animals are behaviorally rhythmic (see Fig 1C)? Doesn't this defeat the purpose of the experiment, which is to correlate behavioral and molecular rhythmicity?  Also, in LD, there is the confound of light-sensitive gene expression.  Studying the transcriptome in DD would have made much more sense.

*We deliberately decided for sampling in LD as these conditions are much more significant for these animals in respect to their natural habitat and our aim was to better understand the general molecular similarities and differences in behaviorally rhythmic vs. arrhythmic animals, irrespective of whether these were under direct circadian clock control or not. The latter aspect might certainly be interesting for future studies.*

*While there will certainly be light-induced gene expression under LD present, both behavioral phenotypes experienced the same light regime before/during sampling for RNAseq and still showed strongly contrasting transcriptomic rhythmicities and GO-term enrichments. While we do not distinguish between transcript cycling driven by the circadian clock or by light, the assemblage of transcripts in which 24h cycling is evoked (whether by the clock or by light) clearly differs between phenotypes, thereby supporting our proposition of a differential regulation of downstream rhythmic outputs. We added further details on this in the discussion, lines 525 onwards “Transcript cycling patterns suggest contrasting rhythmicity strength in behavior versus physiology…”.*

*As will be outlined in more detail below, the aspect regarding the importance of DD vs. LD for characterization was misleading in this context and was changed. It is important to note that to be characterized as either rhythmic or arrhythmic, worms had to show appropriate phenotypes on both LD and DD, as was verified with periodogram analysis and visually. This means that LD rhythmicity of worms characterized as rhythmic was also supported by DD rhythmicity and thus not just a driving light-dependent rhythmicity of an otherwise arrhythmic worms. For arrhythmic worms, this ensured that LD was not masking locomotor behavior. We refer to this now specifically in lines 542-555: “Another aspect that could have affected transcript cycling is the…”*

Second, the authors are quite vague in their description of the method to call animals as rhythmic or arrhythmic.  They say that "For the characterization [of rhythmicity], rhythmicity in DD was considered more important." (page 53, line 591-2), I am not sure what this actually means.  Objective criteria should be used. In addition, as mentioned above, most arrhythmic animals seem hypoactive. This again brings a potential confound, are the authors comparing active vs inactive animals, rather than rhythmic vs arrhythmic? Finally, the issue of whether behavior is really reproducible in individual animals, discussed under #2, needs to addressed since the authors rely on this behavioral reproducibility for their RNAseq studies.

*As outlined in detail above, we now provide very clear evidence that worms can be grouped based on their behavioral rhythmicity by two statistical methods and visual inspection. As to the exact methodology by which worms were sorted for RNAseq analysis, we now also provide a more detailed description of our characterization procedure of rhythmic, arrhythmic or intermediate worms for RNAseq in the M&M section: lines 722-777: “Rhythmicity characterization of wildtype worms and sampling for RNASeq analysis”. The higher importance of DD vs. LD for the characterization was poorly phrased in the initial version of the manuscripts and referred to the fact that e.g. worms with a total of 4 nocturnal activity peaks (over the 6 days of recording) had a higher chance of being identified as rhythmic, if these peaks occurred mostly during the DD phase. To avoid further confusion, we removed this aspect form the text after verifying that the description is in line with our characterization in S6 Fig.*

5.	Regarding the RNAseq results, a critical piece of information to interpreting this data is restricted to a supplemental text (S1 text). I am not sure why this was done this way, but this paragraph definitely belongs to the main text.  Indeed, it explains how low amplitude cycles could occur in the different cohorts, but also confirm the impression from the heat maps that many of the transcripts considered as cohort-specific actually might be rhythmic in both cohorts, but that in one of them amplitude was lower and did not reach significance. It is therefore important to confirm for at least 5 transcripts considered as specifically rhythmic in arrhythmic animals and 5 transcripts scored as specifically rhythmic in the rhythmic animals that they indeed cycle in one but not in the other cohort (or with lower amplitude) using qPCR.  Indeed, I am concerned that lack of statistical cycling in one cohort might just reflect variability in the transcriptomic dataset. A striking example is found with one of the MMPs.  Pdu-MMP7 seems quite rhythmic in both populations, with identical phase - statistical significance is only missed by a hair in the rhythmic animals.  MMPs might be a good case study for qPCR confirmation.

*Upon discussion with colleagues we had been given the advice that the relatively extensive discussion on desynchronization of individuals versus generally low amplitude which can both result in low overall amplitude in the sampling over time was an aspect that could distract from the red thread of the main story. This is why we had originally moved it to a supplementary text. Upon the comment of this reviewer and also upon re-writing the text to improve the general understandability, we now include its major aspects in the main manuscript results part: lines 313-330: “In principle, differences between phenotypes could arise from…” and 384-401: “We next used the identified distinct GO-term groups..”.*

*Upon this comment of the reviewer we also carefully revisited our own heatmap graphs. We had initially chosen the to depict individual graphs of* mmp*s, because we found them potentially particularly interesting in the context of the behavioral changes, but upon revisiting we noticed that they are not very representative of the actual cycling differences that occur. In order to follow our argument on this, we would also kindly ask the reviewer to zoom into the heatmap patterns and then just follow the lines of transcripts that are rhythmic in one behavioral phenotypic group and not the other. There are many examples visible in the heatmap that show that it is not simply a differential amplitude issue. In order to illustrate this better, we now randomly chose several examples and now depict their graphs for better illustration in S8 Fig. Additionally, we also added a table, which directly compares RAIN p-values in rhythmic vs. arrhythmic worms or all transcripts cycling in only one phenotype (S2G Table- please note that this table has to be downloaded from the dropbox link provided at the beginning of the response letter).*

*Regarding the* mmps, *we understand the reviewer’s concern that due to their mainly amplitude differences, but relatively similar overall temporal patterns the differences might not necessarily be as clear as we initially though and would certainly benefit from additional verification work (qPCR and/or HCRs). However, given that any of these additional tests implies an additional full repeat of the entire behavioral characterization and subsequent sampling over multiple timepoints this would likely require another year of work for just getting sufficient animals of the respective behaviorally rhythmic vs. arrhythmic phenotypes. We have relatively high throughput behavioral set-ups, but this is not at the throughput level of those for Drosophila. Furthermore, some animals will mature between behavioral characterization and sampling, meaning that they are lost for RNAseq. We further have to limit behavioral characterization to the phase around new moon to avoid lunar cycle effects, strongly reducing the number of worms that can be characterized per month. The detailed characterization of* mmps *is an interesting aspect, but goes beyond the scope of this manuscript. Given this effort, with what we believe goes well beyond the scope of this manuscript and will only be of limited benefit otherwise (see also paragraph below), we rather decided to remove the details on the* mmps *from the text. In general, we toned down our discussion of mmp patterns and now merely state that, in the context of their role in* Drosophila*, they are interesting targets for future studies (lines 371-378:”* *A check of the associated transcripts…”).*

*As to the general validity of the transcriptomic comparison between behavioral arrhythmic vs. rhythmic worms, the fact that we are able to identify distinct groups or GO-terms distinctly and significantly associated with either rhythmic vs. arrhythmic worms, while by the same time transcripts cycling for both behavioral groups were enriched for core circadian clock components strongly supports our interpretation that the distinctly cycling transcripts in behaviorally arrhythmic worms are not just due to random noise. For better clarification we also added this notion to the result text (lines 379-383: “The identification of distinct groups or GO-terms either associated with rhythmic…”).*

*Finally, we would like to emphasize that a similar observation that behavioral arrhythmicity is not necessarily represented by neither the amount nor the type of transcript cycling has been made in the sea anemone* Nematostella vectensis *(doi: 10.7554/eLife.81084). The results of this study were added to our discussion (lines 556-565: “Of note, a study under sensory temporal conflict conditions…”).*

*Furthermore, evidence that behavioral circadian/diel rhythmicity can be decoupled from the core circadian clock function (while maintaining a functional oscillator) has also been provided by several other studies, e.g. in flies and birds. We now included this in our introduction, see lines 97- 105: “Of note, some drosophilid fly species that inhabit high…”*

*This further emphasizes that our observations are not as unusual as they might seem to be at first glance. Rather, there seems to be a lack of other studies exploring individual & multi-level organismic rhythmicity that we could compare our findings to.*

6.	The authors used the VIO strain to generate their PDF mutant. While the conclusion that PDF is not required for rhythmicity seems solid, how much the VIO controls on figure S8 *(note by authors: now S11)* differ from the VIO results shown on figure 1 is puzzling. What is the explanation for this? Are the differences shown in figure 1 really meaningful if the same strain can differ so much across experiments?

*We agree with the reviewer that this is an interesting notion. Overall, we think that the investigation of marine invertebrates is likely venturing in realms of individual variabilities that are difficult to compare to the standard data typically acquired from terrestrial model systems. Work on the sea urchin genome* Strongylocentrotus pupuratus *showed that the maternal and paternal copies of the genome of ONE individual can differ by 4-5% (doi: 10.1126/science.1133609), dwarfing levels of molecular differences in mammals across species boundaries (humans vs. chimpanzees: ~2%). Such high polymorphism rates have meanwhile been reported for several other marine invertebrates, and, consistently, we also see them in* Platynereis dumerilii*. Even high inbreeding will only slowly reduce these levels. They will have their consequences on output level, including locomotor behavior. We think that is what can be reflected on individual level. The results in Fig 1 on strain level are nevertheless meaningful, because all types of individuals (and their behaviors) are included in these analyses and should thus be representative of the strains.*

Minor comments:

7.	Line 48. Remove "on" from "pdf impacts on overall activity levels"

*Removed as suggested.*

8.	I do not understand the sentence on line 50-51.

*The respective sentence was rephrased to better clarify that this refers to the observed rhythmic phenotypes that are not just clearly rhythmic or arrhythmic but also everything in between, as shown by both behavior as well as the amplitude differences in the RNAseq data (lines 50-52: “Across the* Platynerei*s population, rhythmic phenotypes…”).*

9.	Line 53, linking climate change here seems to be a bit too much of a stretch, particularly in an abstract.  Line 54, this segment of the sentence does not fit with what precedes.

*The second half of the sentence was removed as we understand that the reviewer might see this as too much of a stretch. Concerning the first half of the sentence, it is targeted to the audience of ecologists that currently often do not consider chronobiology as part of their studies and models. It is meant to point out the potential general relevance of the work to raise awareness even to audiences that are less familiar with the topic. We do however recognize that we might have been to extensive on this, which is why we adjusted the sentence accordingly (lines 52-54: “We suggest that such diel rhythm breadth is an important biodiversity resource enabling the species….”).*

10.	Line 83-85, page 4: "rhythmicity depends on the localization of circadian core clock genes expression in the brain and clock gene alleles".  This will be quite obscure for most readers.  Examples should be given.

*We rephrased the introduction, including this sentence, see lines 78-105: “Studies in terrestrial organisms have most frequently…”.*

11.	Line 88-89: "Furthermore, behavioral phenotypes including arrhythmicity can also depend on the circadian clock output pathways".  Again, a vague statement that will be difficult for readers to understand.

*We rephrased the introduction, including this sentence, see lines 78-105: “Studies in terrestrial organisms have most frequently…”.*

12.	Line 90, page 4: "rhythm power:.  Explain what this means.

*The sentence was expanded to include a (brief) definition of rhythm power, lines 93-94: ”These results fit the common assumption…”.*

13.	Line 91, page 4: "is representative for the rhythmicity" should be changed to "is representative of the rhythmicity"

*Changed as suggested.*

14.	Line 105, page 5: "molecularly slowly evolving".  What do the authors mean, and how is this relevant?

*The term “slow molecular evolution” is not uncommon among evolutionary biologists, recognizing the fact that sequences changes with different pace depending on the adaptive pressure species are exposed to. Genomic and transcriptomic analyses showed that* Platynereis *has maintained a rather ancestral exon/intron structure and slow sequence evolution (Raible et al. 2005, Science). In terms of molecular evolution, this annelid is slower than e.g. nematodes and drosophilids, but similar to humans (Raible et al. 2005, Science). This gets particularly relevant when considering why there might be differences in the findings between these different invertebrate model systems and how relevant findings in the annelid worm might be from a larger evolutionary perspective. This is why we think it is relevant and justified to mention the aspect (lines 118-120: “The marine polychaete Platynereis dumerilii is a molecularly…”).*

15.	I would suggest adding a supplemental table summarizing the period, power and %rhythmicity that were observed in the different strains.

*A supplemental table was added (S1 Table), containing period/power values as suggested. ActogramJ does not calculate %rhythmicity and thus the respective information could not be included.*

16.	Line 131-132, page 6: "This suggests that in *Platynereis* the level of behavioral versus metabolic rhythm power is controlled by genes downstream of the core circadian clock." This conclusion cannot be drawn, since the transcriptome data comes from animals in LD that are actively experiencing environmental cues that might be directly driving both behavioral and metabolic rhythmicity. The authors should also acknowledge that they only look for transcriptional changes in rhythmicity, but at the protein level, there might additional regulatory mechanisms.

*We fully agree with the reviewer that as with every gene expression study this work only provides an insight on one important process and post-transcriptional mechanisms can certainly contribute to the observed phenotypic differences. We thus added a sentence highlighting the benefit of adding a proteomic component in future studies: lines 583-585:* “*Ideally, such studies should also include …”.*

*However, we think that our GO-term enrichment analysis is based on different transcripts encoding proteins involved in the same biological processes showing the same patterns. That such overarching patterns do not translate into the protein level differences seems unlikely.*

*We respectfully disagree with the general critique of the reviewer that we cannot state that “in* Platynereis *the level of behavioral versus metabolic rhythm power is controlled by genes downstream of the core circadian clock." While it is correct that the transcriptomic analyses were performed under LD and we thus do not only test for circadian clock effects (by purpose, see our response to comment 4 above), we clearly show that the transcript cycling of all known core circadian clock genes are basically identical. We do not see how this could then result in transcriptional differences of downstream targets (metabolic vs. behavioral/nervous system-related) genes by differences in core circadian clock genes. Let’s assume that the LD cycle had a differential, post-transcriptional, effect on any given core circadian clock gene. As the circadian core clock is a feedback loop, this should also get visible in the transcript cycling of the core clock genes themselves. Given that we don’t see any hint of difference in core circadian transcript cycling, we do not see a reasonable explanation how the circadian clock could be responsible for the differences in the cycling of the other transcripts.*

*We might not be able to fully exclude that light has a differential effect of post-transcriptional processes that could impact on the circadian core clock itself and then subsequently result in the observed phenotypes, but again, it is difficult to image how this should not affect the transcript levels of the core clock genes themselves.*

*The possibility that different phenotypes in LD are due to masking under does not fit with the contrasting behavioral patterns that persisted even though all worms experienced the same LD and DD conditions. We have previously shown multiple time that* Platynereis *locomotor behavior is clock-controlled (it persists in DD), e.g. doi: 10.1016/j.celrep.2013.08.031//* *doi: 10.3389/fphys.2019.00900.*

*It is important to note that worms had to show consistent behavior in LD and DD to be characterized as rhythmic or arrhythmic. Hence, independent of whether transcript cycling in driven by the clock or by environmental factors (light), it is clearly phenotype-specific. As we see no differences in the circadian clock (see above), differences in rhythmic phenotypes must emerge downstream of the clock, whether driven primarily by the clock itself or by phenotype-specific environmental modulation. We added a section to address this aspect: lines 542-546: “Another aspect that could have affected transcript cycling…”.*

17.	Line 158, page 7: there appears to be more diversity between strains under LD than under DD - could this represent strain-specific masking behavior?

*While this is possible, inter-individual diversity is also larger in LD (see Fig 1C,D) and in all strains there were both highly rhythmic and completely arrhythmic individuals. Thus, this observation is rather related to the overall decrease in rhythmicity in DD, with the loss of bimodality in the VIO strain being the result of the peaks associated with lights off/on in LD now drifting in free-run. Please also see last paragraph of response to comment 16.*

18.	Line 266-67, page 10: "This highlights that behavioral rhythmicity and metabolic/physiological rhythmicity do not necessarily correlate and can in fact be opposite."  Similar issues as in minor comments #9.

*We are unsure what the reviewer is referring to. Comment 9 (now re-numbered as comment 15) suggested the addition of a supplemental table and we don’t see how this could apply to the marked sentence. In case this is supposed to refer to possible differences between gene expression and protein level, please see response to comment 16.*

19.	Line 279-281, page 11: neither the distribution of rhythmic MMP transcripts nor the cycling of them is identified in Figure 3 - that figure should not be cited here.

*In the process of restructuring the mmp aspects (see response to comment 5), the respective part was completely removed.*

20.	Line 318-319, page 12: Does any PDF antibody work in *Platynereis*?  It would be great to confirm absence of expression by IHC.

*As suggested by the reviewer, we performed PDF IHC in wildtype and two independently generated mutant worm strains and confirmed the absence of PDF in both mutant alleles, as well as transheterozygous individuals. The respective pictures were added as S9 Fig, referred to in lines 431-434: “Both alleles are predicted to…” and 480-484: ”Together, the results suggest that while PDF controls…” and the description of the custom-generated antibody and IHC protocol was added to the M&M section (lines 915-920: “Generation of anti-Pdu-PDF antibody…”.*

21.	Line 333-338, page 13: were the animals used for these experiments mutants from the original VIO background, or from the backcrossed VIO/PIN background?

*The animals were from the original VIO background. This information was added to the text (lines 448-450:”* *We determined the potential effects…”). As there was no difference in core circadian clock gene cycling, we do not think that this could be influenced by further backcrossing. Previous works showed that the different wildtype strains also do not differ between their core circadian clock cycling (doi: 10.1016/j.celrep.2013.08.031, doi: 10.1007/978-4-431-55261-1_8), and the less backcrossed animals had a more severe rhythmic (non-*pdf-*linked) phenotype than further backcrossed animals.*

22.	Line 382-387: "Differences in the strength of behavioral circadian rhythms were not mirrored by the transcript oscillations of all core circadian clock genes, nor in the number of overall cycling transcripts. Circadian behavioral rhythm differences were, however, mirrored by the types of transcripts showing cyclic expression: neuronal/behavioral versus metabolic/physiological transcripts correlating with behaviorally rhythmic versus arrhythmic worms, respectively." This statement is problematic - it reads as though the behavioral and transcriptional rhythmicity are co-occurring, when the behavioral rhythmicity is mostly determined under DD while the transcriptional rhythmicity was studied under LD.

*We rephrased the respective sentences, lines 502-510: ”Differences in the strength of behavioral rhythms did…”.*

23.	Line 445-46, page 17: wouldn't it be expected for all worms, regardless of behavioral phenotype, to exhibit identical expression patterns for circadian clock genes while in LD conditions? After all, the idea of the authors is that the clock drives circadian rhythms of different pools of genes, and it would be expected to be entrained in a similar manner by the LD cycle. Actually, evidence in Nile grass rats suggest that diurnality/nocturnality is dependent on outputs, not on the phase of the circadian oscillator (see for example Lambert 2005, JBR).

*We respectfully disagree that core circadian transcripts patterns should be necessarily expected to be identical under LD. In fact, it is well established from studies of human chronotypes that behavioral differences have their molecular counterparts in period lengths difference of the circadian core clock (which translates into phase difference under entrainment), e.g. see* *doi: 10.1073/pnas.0707772105. Similarly, our own work on the diel emergence timing of the marine midge* Clunio *revealed difference in core circadian clock period lengths (again translating into phase difference under entrainment): see doi: 10.1038/nature20151.*

*In dipteran flies it has been prominently proposed that differences in the strength of the circadian core clock represent adaptations to latitudes under entrained conditions, e.g. see doi: 10.1016/j.cub.2019.09.032 and references therein.*

*Similarly in reindeers, e.g. see doi: 10.1007/s00114-006-0174-2, doi: 10.1016/j.cub.2010.01.042.*

*Thus, from the available literature the question if or if not behavioral rhythmicity vs. arrhythmicity is reflected on the level of core circadian clock transcript cycling was an open question. We knew from multiple previous studies that* Platynereis *locomotor activity is under the control of a (plastic) circadian-circalunidian clock (see recent study doi: 10.1073/pnas.2115725119) and we know that this behavioral plasticity is reflected in phase shifts and amplitude of core clock transcripts, such as* per *under the respective moonlight/sunlight/darkness conditions (doi: 10.1016/j.celrep.2013.08.031 and Zurl et al, unpublished). In our opinion it could have thus been well possible that the oscillations of core circadian transcripts are effected, even under LD.*

*We expanded the introduction to make this point clearer and included additional (above mentioned) scientific studies in this, see lines 78-105 “Studies in terrestrial organisms have most frequently identified changes to the core…”.*

*We also partially rephrased and expanded the part to address the cycling of clock genes in LD, lines 542-555: ”Another aspect that could have affected transcript cycling is the exposure to LD cycles…”.*

*Regarding the aspect that rhythmic phenotypes are shaped downstream of the circadian clock, please also see in addition our response to comment 16.*

24.	Line 483, page 19: should change to "We identify consistent circadian behavioral rhythmic diversity"

*Changed as suggested.*

25.	Line 491, page 19: "Most notably, our observation of enhanced diel cycling of metabolic/physiological transcripts in behaviorally arrhythmic worms" again implies that the behavioral and transcriptional rhythms are co-occurring. Worms were much more behaviorally rhythmic in LD than in DD.

*In this sentence we clearly refer to diel rhythmicity, i.e. under LD. And under these environmental conditions we do expect the behavioral and transcriptional rhythms to co-occur. After all, to be characterized as e.g. arrhythmic, worms had to show the appropriate behavior in both LD and DD. Please also see response to comment 4.*

26.	Figure S5 (now S8C,D): why only include transcripts associated with GO-terms?

*A complete visualization as done in S8C,D Fig but with all differentially cycling transcripts resulted in graphs too crowded to still identify pairs of rhythmic/arrhythmic SD data points. It would be unreadable. We focused on the mentioned transcripts as they are the ones most representative for the respective phenotypes and thus the ones most relevant for our reasoning.*

**Reviewer #3**:

Behavioral rhythms are bad predictors of general organismal rhythmicity

1. The manuscript by Häfker et al. describes their study of circadian rhythms in the marine worm *Platynereis* *dumerilii*. They discovered substantial variation between individual worms, which was repeatable. They focused mainly on the power of rhythm and identified rhythmic and non-rhythmic worms. Intriguingly, a transcriptomic survey revealed that the core circadian clock is intact in arhythmic worms. In addition, analysis of non-circadian cycling transcripts revealed a general difference between rhythmic/ rhythmic worms: neural/behavior vs metabolism/physiology-related, respectively. Lastly, the study demonstrates that PDF does not serve as an output gene in *Nereis*, in contrast to its role in insects. Overall, this is an interesting study, data-rich, which uncovers several intriguing phenomena and underscores the importance of individual behavior.

*We would like to thank the reviewer for the overall positive assessment of our work.*

2. There are however several issues with this study that need to be addressed. First, minor as it may sound, there is an issue with the title, which reads as a universal overarching statement about behavior being "bad predictor for organismal rhythmicity".  This is definitely not the case in many animals (insects and mammals), where mutagenesis screens based on behavioral analysis led to the identification of circadian clock genes. At best, behavior is a bad predictor for rhythmicity in *Nereis*, but further work needs to be done in order to extend this statement to other organisms.

*This is certainly a relevant point that also led to significant discussions within the group. Of note, this point was also raised by reviewer #2. We modified the title to:*

*“Behaviorally rhythmic and arrhythmic individuals of a marine annelid exhibit similar levels of molecular rhythmicity in the brain”*

*Concerning the potential general significance of our finding it should be noticed, however, that in a recent study of sensory conflicting environmental cues, the behaviors of the sea anemone* Nematostella vectensis *became arrythmic, but highly consistent with our study, this was not reflected on the transcript level. We have included this recent study in our discussion section (doi: 10.7554/eLife.81084), see lines 556-565: “Of note, a study under sensory temporal conflict conditions in the sea anemone* Nematostella vectensis…”*.*

*The fact that behavior rhythmicity was possible to be used as read-out for genetic screens does not necessarily imply that it is a faithful predictor for other rhythms in the organisms’ body either. The fact that such other (non-behavioral) circadian/diel rhythms exist has been very well established also for several organs in the mammalian body (liver, kidneys, skin fibroblasts). How well these other processes are aligned with behavior as read-out is not well studied. Furthermore, several studies already suggest that ~24h behavioral and non-behavioral processes are modulated downstream of the core circadian clock. We refer to some examples now also in our introduction, see lines 97-105: “Of note, some drosophilid fly species that inhabit high latitudes become … .”*

*However, nevertheless especially scientists starting to look into the chronobiology of non-conventional model systems (but even some colleagues on conventional models, such as Drosophila) often use behavior as the only read-out to then state if or if not THE ANIMAL is rhythmic, has a strong or weak clock etc. Our aim is to emphasize to these colleagues that behavior is exactly just one read-out and as such it can be very misleading to base assumption on e.g. circadian clock strength or metabolism on this read-out. This is why we think that our study is clearly of general relevance.*

3. Close analysis of the actograms in Figure 1 indicates that that the rhythmicity is not obviously different between VIO and the other strains. However, what is clearly different are the duration of the activity bouts and the shape of the distribution. It is clear the behavior of VIO is bimodal, while the other strains are unimodal. These differences in the shape of the waveform will affect the spectral analysis and will lead to false differences in the rhythm power, although the actual rhythmicity is identical.

In addition, in some of the actograms, there is very little amount of activity (Fig 1E, Fig S2, A3). These individuals clearly need to be excluded from the analysis. I would also guess that these individuals are not that healthy.

Overall, the individual differences that might be observed, in my opinion, reflect differences in the level of activity, which might be interesting but is not related to the circadian system.

*A partly overlapping comment was also made by reviewer #2 (comment 2), especially concerning the sick animals. As this point was raised by two reviewers, we apologize that this point was apparently not made sufficiently clear, but of course we exclude any even potentially sick animal from our analyses. For any of our experiments we do of course check the worms for their health. We have no indication that these worms were in any way less healthy than more active/rhythmic ones. All worms used for behavior experiments were visually investigated for signs of sickness (twitching, lack of tube-building, changed color, inclusions/ulcers) and were only used, if there were no abnormalities. We now included this aspect in detail in the manuscript: lines 641-643: “All worms were inspected for signs…”, lines 747-758: “To further ensure that the supposed inactivity of arrhythmic …”*

*Furthermore, it is not uncommon in circadian clock mutants in mice and Drosophila to have overall lower activity, e.g. bmal -/- mice*: [doi: 10.1371/journal.pone.0138661,](doi:%2010.1371/journal.pone.0138661,) *cwo -/- flies:* [*doi: 10.1177/0748730407313817*](doi:%2010.1177/0748730407313817). There is nothing “sick” about this phenotype in general.

*An additional point that argues against the investigated arrhythmic worms being in any way “unusual” or sick: In the group of worms characterized for RNAseq analysis and maintained for sampling, we saw that in the time between behavioral characterization and sampling (~1 month) maturations and spawnings occurred in both rhythmic and arrhythmic worms at the same lunar phase, over the same duration of the maturation period and with the same reproductive success. Swimming speed during their mating dances was indistinguishable. This aspect is now also mentioned in the manuscript: lines 754-758: “*Additionally, as some worms matured between…”

*In summary, there is no indication that behaviorally arrhythmic worms have a lower fitness than others and hence there is no reason to not consider or question these worms.*

*Concerning the aspect that the differences rather reflect levels of activity, than circadian differences:*

*It appears to us that this reviewer is under the impression that our animals didn’t really move anymore. This is, however, incorrect. In short words, it’s a Y-axis issue. The worms show movements, and irregular movements across time can clearly be called arrhythmic.*

*As a longer explanation: This impression probably occurred, because we should have illustrated better that the arrhythmic worms are actually not inactive, but merely lack a nocturnal increase in locomotor activity. The existing lower, but not temporally “bundled” levels of activity are often not very well visible when the focus of the thresholds of the activity plots is on the activity bouts. To show baseline activity more clearly, high activity bouts would have to be cut off and then those would be neither properly quantifiable nor comparable across worms, which is why we usually set the thresholds in these activity diagrams the way we did.*

*To better illustrate this point, we added two supplemental figures (S4 Fig and S7 Fig) that re-plots all worms characterized as arrhythmic and seemingly lacking activity from Fig 1, S2 Fig* and *S7 Fig with a 10-fold scale increase of the y-axis.*

*For further illustration, in S7B,C Fig we also show actograms of worms that crawled out of the tracking wells to illustrate the stark difference between an arrhythmic worm that is moving regularly and background tracking noise.*

*It is important to clarify that rhythmic and arrhythmic worms did not generally differ in their activity during daytime (LD) or prospective daytime (DD). Additionally, it is important to emphasize that arrhythmic worms were no an outlier group, but part of a behavioral continuum with rhythmic and arrhythmic worms at the ends and various intermediate phenotypes in between. Thus, we think that the worms with randomly distributed activities across time can clearly be called arrhythmic and our interpretations are justified.*

4. At the evolutionary level, the authors argue that circadian phonotypic diversity will expand the ecological niche of the species, but this is usually true to the phase of the circadian rhythm, not the power of the rhythm. It was demonstrated several times that under fluctuating environments, rhythmic behavior contributes to fitness. So, it is hard to understand how arhythmic behavior will serve an adaptive function (the only example I know is of nursing honeybees that work around the clock in the hive).

*Firstly, we would like thank the reviewer for reminding us of the bee hive example, which clearly shows that behavioral arrhythmicity vs. rhythmicity can in fact be subject to evolutionary selection. We now also cite corresponding work in our manuscript, lines 538-541: “…as well as in individual clock neuron groups of forager and…”.*

*However, the bee hives are not the only example. There is quite some literature about “strong” vs. “weak” circadian rhythms/oscillators and how this might be beneficial to species, depending on latitude, e.g. see for insects: doi: 10.1177/074873049100600402, doi: 10.1016/j.cub.2017.01.036 and doi: 10.1016/j.cub.2019.09.032. The last paper was already quoted as ref 17, now ref 20 in the introduction of our manuscript, which we now also expanded, lines 97-105: “Of note, some drosophilid fly species that…”.*

*Further examples:*

*for zooplankton: doi: 10.4319/lo.2010.55.2.0831, doi: 10.1098/rsos.230520.*

*for mammals: doi: 10.1152/physiol.00045.2014, doi: 10.1007/s00114-006-0174-2.*

*The comment of the reviewer let us, however, realize that we should not be too speculative on the reasons for the observed rhythmicity/arrhythmicity differences and thus shortened the discussion about it significantly. It likely represents different life strategies, but the meaning of those in the local seagrass meadow environment needs to be tested, which certainly goes beyond the focus of this manuscript. We provide a first layer of speculation as possible direction for future studies in the (significantly shortened) discussion, see lines 577-585: “Platynereis live in heterogeneous coastal habitats and practices broadcast spawning where planktonic larvae get…”, see also response to comment 27.*

*For entire populations, we would suggest that the benefit does not arise from arrhythmicity in itself, but from the diversity of rhythmic phenotypes (our study focusses on the extremes, but as we state- in population this represents a phenotypic continuum) that increase chances that at least some offspring will survive by showing more or less temporally focused exploratory behaviors under unpredictable conditions (bet-hedging, doi: 10.1007/s12038-015-9506-8, doi: 10.1146/annurev-ento-120220-101047). It might also be a way for the species to diversify food resource usage and to reduce intraspecific competition. The former can get especially relevant when environmental conditions change.*

*In general, (rhythmic) phenotypic diversity is beneficial in heterogeneous habitats where there is no single “optimal fitness phenotype” and in cases where organisms have limited ability to actively select their habitat (to make it match their respective phenotype). Both of these points apply to* Platynereis *that inhabits heterogeneous coastal areas and practices broadcast spawning where offspring get dispersed by ocean currents.*

5. I find it a bit odd that the fact that *Platynereis* has been a major model for studying circalunar rhythms (by the authors) is not receiving any discussion in the paper. Perhaps the intriguing pattern of cycling expression in these worms relates to the fact that the worm navigates between two different pacemakers.

*Again, we apologize that we did not explain this better in the original text.*

*While the lab also works on the interactions between different timing systems, specifically circalunar and circadian(/circalunidian) timing, this specific study aimed to provide a better understanding for the variability that we have been noticing when performing our circadian(/circalunidian) locomotor experiments. Thus, this study is entirely focused on circadian locomotor aspect from the onset. Therefore, all worms in the study were behaviorally characterized and/or sampled at the same time of the lunar cycle, around new moon. As indicated in the original manuscript’s method section, we specifically chose* *the time around new moon, which is the time when the impact of moonlight and the circalunar clock on the circadian(/circalunidian clock is absent. See doi: 10.1073/pnas.2115725119 and doi: 10.1016/j.celrep.2013.08.031 for more background on this*. *Thus, by the whole study design, we excluded the circalunar (and moonlight) effects to the best of our knowledge. But we agree that we could have pointed this out more clearly early on and thus we now also added more background on this in the results section, see lines 156- 161: “In our previous studies we repeatedly observed a high diversity…” and lines 272-274: “To prevent possible effects of Platynereis…”.*

Minor comments:

6. line 132-133, I don't understand this sentence, PDF is also an output gene

*There should have been a paragraph break between the sentence before and the sentence about PDF. We rearranged the sections referring to clock, behavioral and metabolic rhythms, as well as the section referring to PDF to clearly set them apart, see lines 136-147: ”In contrast, cycling of core circadian… “.*

7. line 152. Please clarify what is "non-incrossed closed culture"

*We rephrased the respective sentence (lines 164—167:”* *We chose three worm strains…”). The term “closed culture” refers to no further genetic input by adding/crossing animals from outside this culture itself.*

8. line 154, should be Fig S1

*According to the PLoSBio guidelines, supplemental items are supposed to be shown like this (S… Fig & S… Table) and hence the order was kept. Maybe this is thought to provide a better distinction from the main manuscript figures? We did however change “Tab” to “Table” throughout the manuscript as suggested in comment 21.*

9. line 171- figure 1 legend, scoring arrhythmicity based on visual actogram inspection is not a reproducible method

*We apologize, this was not well phrased and removed the respective lines. We now also provide a more detailed description of our characterization procedure for RNAseq in the M&M section, lines 722-777 “Rhythmicity characterization of wildtype worms and sampling for RNASeq analysis…”. Please also see our detailed response to the request for statistics in comment 11 below.*

10. line 179: I don't understand "due to maturation of the other individuals", please clarify

*The sentence was rephrased to clarify that this refers to animals that matured before a repetition run could be performed. To further explain, when* Platynereis dumerilii *mature, they don’t just change their physiology and body color (well discriminable), but also their behavior. This is why any animal that shows the body color signs of maturation got excluded from the analyses. See lines 203-204: “Repetitions were limited, because…”*

11. line 181, the comparisons of the first and second run should be carried out in a quantitative manner, using some sort of statistics

*As this point was also raised by reviewer #2, we put significant time investment into improving our analyses method to be more quantitative. We implemented two fully independent statistical approaches, which differ in the amount of complexity reduction of the data, to ensure that such reductions (which have to be done to some extent in any dataset) are not influencing the overall result. Both of our analyses show that repetition runs from the same, matching worms are significantly more similar in their behavioral profile, compared to the repetition runs of other, non-matching worms.*

*We first used are binary activity overlap approach, where the activity profile of each worm behavior run was transformed into a binary activity vector and then compared with the repeat run and the mean of all activity scores of all other repeat runs. The second approach is based on an already published statistical comparison method for locomotion similarity of behavioral patterns in the marine crab Uca (doi: 10.1371/journal.pone.0175403). It employs Kolmogorov-Smirnov tests to compare behavioral patterns between matching initial/repetition runs as well as to all other (non-matching repetition runs). The resulting p-values from each matching initial/repetition-comparison are treated as proxies of similarity and were compared to the remaining (non-matching) repetition runs via single-sample Wilcoxon signed-rank tests.*

*The results of the first, binary approach are now visualized in S5 Fig, while the results of the second approach were added to the initial/repetition actograms in Fig 1E and S3 Fig as scatter plots next to the respective actograms.*

*Importantly, both methods fully confirm our original statement of a higher similarity of repeat runs from the same individual than repeat runs from other individuals, especially for PIN strain worms. Both quantitative behavioral methods also support that behavioral rhythmicity vs. arrhythmicity is a non-random behavioral feature of individual worms. We believe that this forms as solid basis for our subsequent sampling for molecular analyses.*

*The description of the analysis was added in lines 212-253****:*** *“In addition to visual inspection of initial/repetition similarity, we…” and lines 699- 720: “Similarity analyses of individual worm behavioral patterns…”*

*Importantly, while we agree that quantitative behavioral approaches were missing and needed, we also want to point out the risk of reducing complex behavioral patterns by any quantitative method. Hence, in addition we also always ensured any conclusion by visual verifications.*

12. line 224: More important than the fact that similar number of transcripts cycle in rhythmic and non -rhythmic animals, is to what extent these are the same transcripts

*We agree with the reviewer and expanded the sentence to state that the overlap in cycling transcripts was limited and what these are, lines 303-312: ”* *For the core circadian clock and neuropeptide transcripts, RAIN analyses identified… ”, lines 353-378 “Cycling transcripts shared by worms of both phenotypes showed…”, as well as Fig.3B-D and S3 Table.*

13. line 228: reword "kinetics

*Changed to “temporal expression patterns” (lines 305/306).*

14. line 230, I am not sure about the meaning of "significant common cycling

*The word “common” was removed to avoid confusion.*

15. line 237 delete "RAIN-significant (here and everywhere else)

*Everywhere removed as suggested.*

16. line 267. I don't understand what is the evidence for that statement (that the two types of rhythmicity oppose each other)

*We agree that this was poorly phrased and replaced “opposite” with “highly contrasting”. It refers to the differential enrichment of GO-terms in behaviorally rhythmic and arrhythmic worms, see lines 366-368: “This highlights that behavioral rhythmicity…”.*

17. line 268: if the oscillation amplitude of the transcript is informative, please describe the effect in the main text

*Upon discussion with colleagues we had been given the advice that the relatively extensive discussion on desynchronization of individuals versus generally low/high amplitudes (which can both result in low overall amplitude in the sampling over time) was an aspect that could distract from the red thread of the main story. This is why we had originally moved it to a supplementary text. Upon the comments of this reviewer and reviewer #2, and also upon re-writing the text to improve the general understandability, we now include its major aspects in the main manuscript result part (lines 313-330: “In principle, differences between phenotypes could arise from…” and 384-341: “We next used the identified distinct GO-term groups..”).*

18. line 278, for the sake of reproducibility, please provide the evidence, rather than citing personal communication.

*We strongly shortened the section on the mmps from the manuscripts to focus the story line on our most well-founded observations. Thus, also the “pers.comm” reference is removed from the text.*

19. line 279, I don't understand the text.

*As mentioned above, the respective section on the mmps was removed.*

20. line 280 delete "RAIN-

*Removed as suggested.*

21. line 358, change to Table S7 (here, and all other supp. tables)

*Done.*

22. line 356. I don't understand the logic here. The fact that PDF is not essential for the rhythm does not preclude the role of MMPs that might be driven by other proteins than PDF.

*We assume the reviewer is referring to line 365 instead of 356 in the original manuscript version (typo?), as there is no mentioning of MMPs at line 356 or those above/below. Line 365 was removed, see comments above.*

23. Line 379, I am not clear about the role of "However" here. as it is not related to the previous sentence. Also, starting two consecutive sentences with 'however'" is problematic.

*Changed to “Furthermore,”*

24. line 384: I don't think that the actual number of cycling transcripts is that important.

*We agree, we just mention the absolute numbers for a better understanding where our calculations about the relative fraction of changing transcripts (from the total transcriptome) come from. To clarify this better, we kept the numbers in the results part, but rephrased the sentence accordingly in the discussion, lines 502-504: “Differences in the strength of behavioral rhythms did…”*

25. line 408. diversity in circadian phenotypes should be manifested in phase variation, which has not been analyzed here. If strong rhythmicity is useful for fitness, it should have been under stabilizing selection, and have minimal variance.

*We decided to significantly shorten the sections concerning the potential ecological benefits, as these go beyond the first layer of speculation backed by our data. The respective sentence was hence removed.*

26. line 410. The notion that natural selection's role is to benefit the whole species is rather an archaic view.

*We decided to significantly shorten the sections concerning the potential ecological benefits, as these go beyond the first layer of speculation backed by our data. The respective sentence was hence removed. See also response to comment 4.*

27. lines 422-428, in the discussion on bet-hedging it is not clear how specifically rhythmicity/Arrhythmicity can be beneficial at different conditions.

*We decided to significantly shorten the sections concerning the potential ecological benefits, but decided to maintain this first layer of speculation as possible direction for future studies in the (shortened) discussion, see lines 577-585: “Platynereis live in heterogeneous coastal habitats and practices broadcast spawning where planktonic larvae get…”, see also response to comment 4).*

28. line 471, I don't see any reason why cycling genes link to behavior and neuronal plasticity indicate a more explorative lifestyle. the whole discussion in lines 477-480 is overly speculative and is not very convincing.

*We decided to significantly shorten the sections concerning the potential ecological benefits. Thus, this specific sentence was removed, but see also response to comment 4.*

29. lines 489-499 I don't think it was ever argued that behavioral output is an exclusive indicator of circadian rhythmicity.

*Of course, it has never been stated as an exclusive indicator of circadian rhythmicity, however, it is often used as such. How often do papers conclude on overall organismal rhythmicity based on behavioral data alone, e.g. stating that a given individual is rhythmic or arrhythmic?*

*As mentioned above, especially scientists starting to look into the chronobiology of non-conventional model systems often use behavior as the only read-out to then state if or if not THE ANIMAL is rhythmic, has a strong or weak clock etc. Our aim is to emphasize especially to these colleagues that behavior is exactly just one read-out and as such it can be very misleading to base assumption on e.g. circadian clock strength or metabolism on this read-out.*

*By rephrasing several parts of the manuscript, we hope that we have clarified this aspect.*

30. Figure 1A-C The difference in rhythm power in VIO is not that apparent, but its behavior looks bimodal compared to the others that are unimodal. In DD, the activity of VIO is unimodal as the other and there is no significant difference in the rhythm power.

*We agree and added at sentence at the end of the respective paragraph to address the specific activity of VIO worms and its effects of rhythm power. We don’t call it bimodal in the text, because several worms show only one, but short, activity peak, which in sum across all animals looks bimodal, but might not be bimodal on the individual level. But as we agree with the reviewer that the specific way of movement might cause effects on rhythm analyses, we comment about it in the text, see lines 176-178: “For the VIO strain, the lower rhythm power…”.*
