# Supplementary material for: Molecular circadian rhythms are robust in marine annelids lacking rhythmic behavior
Source: PLoS Biol. 2024 Apr 11;22(4):e3002572. doi: 10.1371/journal.pbio.3002572 (PMC11008795; doi:10.1371/journal.pbio.3002572)
Supplement: Attachment — Submitted filename: r-p_PlosBio_rev2_responses to reviewers_2.docx [file pbio.3002572.s021.docx]

*We are thankful for the reviewers’ efforts of evaluating our revised work. We appreciate their recognition of our extensive additional work. Their remaining comments helped us to further sharpen the conclusions in our manuscript.*

**Reviewer #2**:

The authors have extensively revised and improved their very interesting manuscript. My major concerns have been addressed convincingly and thoroughly. I have a few suggestions for the authors to consider:

1. There is no doubt that there are important phenotypic variations between strains on figure 1. However, as I mentioned in my first review, the same strain can behave rather differently over time (S11). Therefore, I wonder whether the variations seen between strains on figure 1 are really strain-specific, or if they reflect, at least in part, the state of the colonies at a specific time. It seems difficult to know for sure at this point, so it would be worth bringing this up in the result or discussion section.

*We agree that it is possible that the behaviors of the strains (although inbred) could still change over time, due to the (random) selection for specific alleles combinations. We now added a paragraph in the discussion that addresses the topic of strain differences and behavioral pattern inheritance (line 564-569: “While our behavioral data suggest some strain-specific characteristics, there…”).*

2. Really striking how, on figure S5, the high reproducible behavior are lost in the control pairing. I had noted in the previous review that it seemed the highly reproducible behaviors were frequently "hypoactive" animals. The authors have now convincingly established these animals are active throughout, but do not show a nocturnal peak of activity, and importantly do not show any sign of sickness. It would be interesting to determine whether indeed this lack of nocturnal behavior is enriched in the most reproducible phenotypes, or perhaps even drive most of the reproducibility. It might be the most stable behavior state. It might also be worth mentioning the supplemental figures magnifying the activity of the arrhythmic, low activity animals earlier in the text.

*As suggested,* *we moved the first mentioning of the Y-axis magnified figures to line 180-182: “Importantly, arrhythmic individuals were not immobile…”.*

*Concerning the aspect of the similarity between the runs was dominated by the arrhythmic individuals, we respectfully disagree. The statistics in Fig 1E, as well as S4 Fig (formerly S3 Fig) show that comparing the similarity scores of an arrhythmic individual to the remaining ones did not reveal any generally higher reproducibility for the arrhythmic worms. Based on this and the fact that marking each data point for e.g. very rhythmic or arrhythmic individuals in S5 Fig would significantly reduce the clarity of the figure, we decided against this.*

3. Line 201: Rephrase : "To closer investigate" to " To investigate more closely ".

*Changed as suggested (line 211).*

4. Line 84-5. The statement here is worded too strongly in my opinion, though the data in rev 15 certainly suggest such a model.

*We toned down the statement by adding a “likely” to the sentence (line 88).*

**Reviewer #3**:

1. The revised manuscript addresses many of the comments that I made on the original version. However, a couple of issues have not been resolved. My major point #3 was that the different patterns of behavior (e.g., uni- vs bimodal profile) may affect the spectral analysis and, therefore, are not comparable. Furthermore, some of the actograms in Figure 1E are just blank graphs with no activity. This low level of activity is not suitable for rhythm analysis.

*We are rather surprised about the comment on Fig 1E. It does not contain any empty actograms, but shows arrhythmic individuals that do not display any major activity peaks. We clearly detailed in the text and also in show in S3 Fig (formerly S4 Fig) and S7 Fig, that these worms are not inactive at all, but arrhythmic. While arrhythmic individuals obviously provide little information in terms of rhythm period, we focused on behavioral patterns for our statistical analyses. Hence there was no reason to not consider them.*

*Concerning the bimodality, as outlined above, for the behavioral individual reproducibility analyses, we analyzed behavioral patterns not the rhythms. Hence, the (bi)modality is not an issue for this analysis. In fact, we would like to point out that the individual worms are not necessarily bimodal, but often show activity bursts at dusk or dawn, which in sum looks bimodal. We addressed the lower rhythm power due to this type of activity distribution already in the previous revision (now lines 182-185: “For the VIO strain, the lower ….”), but do not see how this would further affect the main statements of the manuscript and hence did not elaborate in further detail on it.*

2. The other point that I have raised is about the suggestion that arrhythmic behavior is adaptive because it is beneficial for the species (this suggestion is also present in the abstract). However, selection is driven by individual fitness, not the species.

*In the first revision, we addressed the potential benefits of arrhythmic behavior (now lines 627-632). We now further expand upon this, stating examples of arrhythmic behavior as a way of maximizing food intake, even when the circadian clock itself and metabolic processes stay rhythmic (lines 632-636 “Similar patterns have been observed in Arctic vertebrates that show arrhythmic behavior during the…”).*

*It should also be considered that while selection acts on individuals, bet-hedging can ensure that the genetic information of the parents lives on, even if individual offspring should have lower fitness. Considering the heterogeneous habitat and the planktonic larvae phase of Platynereis, it can further be assumed that ‘optimal’ (circadian rhythmic) phenotypes differ depending on the conditions encountered upon larvae settlement, which allow (and might select for) multiple phenotypes to exist within one population. This scenario is compatible with balancing evolution where multiple genotypes can persist in a population, if they result in phenotypes that all can be beneficial for the respective individual fitness in a heterogeneous environment. We expanded the respective section in the discussion to explain this aspect further (lines 637-653: “*Platynereis *live in heterogeneous coastal habitats…”).*
